# A new perspective on macrophage-targeted drug research: the potential of *KDELR2* in bladder cancer immunotherapy

**DOI:** 10.3389/fimmu.2024.1485109

**Published:** 2024-12-03

**Authors:** Zhiyi Zhao, Hongling Jia, Zhou Sun, Yumeng Li, Lingyun Liu

**Affiliations:** ^1^ Department of Andrology, The First Hospital of Jilin University, Changchun, Jilin, China; ^2^ The First Clinical Medical College, Shandong University of Traditional Chinese Medicine, Jinan, Shandong, China; ^3^ Department of Urology, China-Japan Union Hospital of Jilin University, Changchun, Jilin, China

**Keywords:** bladder cancer, *S100A8*, TCs, *KDELR2*, macrophage

## Abstract

**Introduction:**

Bladder cancer was recognized as one of the most common malignant tumors in the urinary system, and treatment options remained largely limited to conventional surgery, radiotherapy, and chemotherapy, which limited patient benefits.

**Methods:**

Researchers constructed an RNA transcriptome map of bladder cancer by integrating single-cell RNA sequencing and clinical data, identifying potential molecular targets for diagnosis and treatment. We also verified the antitumor activity of the target through in vitro experiment.

**Results:**

A distinct tumor cell subpopulation characterized by elevated *S100A8* expression exhibited high copy number variation, high stemness, and low differentiation. It interacted with myeloid cells via the MIF-(CD74+CD44) and MIF-(CD74+CXCR4) signaling pathways. This study underscored *KDELR2*’s role in promoting cell proliferation, invasion, and migration, providing new therapeutic insights. Prognostic analysis revealed that *KDELR2* correlated with poor survival, higher immune scores, and increased macrophage infiltration.

**Discussion:**

The findings suggested that patients with high *KDELR2* expression might benefit from immune checkpoint therapy. *KDELR2* was also shown to enhance bladder cancer cell proliferation, invasion, and migration, highlighting it as a promising target for macrophage-focused drug development.

## Graphical Abstract

Workflow diagram of this study. Workflow showed bladder cancer single-cell RNA sequencing data were used to perform cell stemness, pseudotime, and cell communication analyses, leading to the identification of a cell subpopulation and the creation of a prognostic model. The *KDELR2* gene was validated by in vitro experiments.

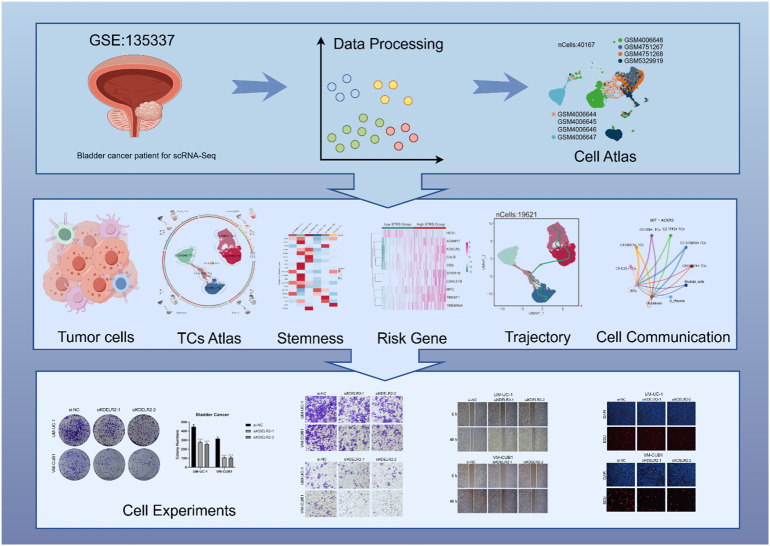

## 1 Introduction

On a global scale, bladder cancer holds the second position among urological malignancies in terms of prevalence, while it ranks ninth among all malignancies and stands as the 13th leading contributor to cancer-related deaths worldwide (1). The main risk factors include old age, smoking, pelvic radiation therapy, the use of cyclophosphamide, and an enlarged prostate and urinary retention in men, which may also increase the risk of cancer due to the presence of carcinogens in the urine (2). Clinically recognized bladder tumors typically present with symptoms such as significant hemorrhage, urinary system irritative symptoms (e.g., urgency, frequency, and burning), and may or may not be accompanied by massive hematuria. This is especially true for patients with diffuse *in situ* carcinoma, muscle-invasive tumors, or secondary infection-related lesions. While some bladder tumors may be asymptomatic, they can be detected during the evaluation of asymptomatic hematuria (3).

Current modalities for bladder cancer management encompass surgery, chemotherapy, and radiation therapy (4). Immunotherapy for bladder cancer had made strides in recent years, but the benefits were limited, particularly in certain types of bladder cancer. Therefore, researchers aimed to develop more precise targeted therapies and combination strategies. The recently advanced single-cell RNA sequencing(scRNA-seq) technology facilitates precise profiling of diverse gene modules utilizing minimal cell numbers, enabling high-resolution characterization.

*KDELR2* is an ER-resident protein involved in the ER stress response and proper protein folding. Its expression in cancer cells could be associated with tumor proliferation, migration, and invasion. For instance, in breast cancer, *KDELR2* was identified as a novel target of HDAC3, with aberrant expression predicting poor outcomes in patients (5). Though *KDELR2*’s role in bladder cancer had not yet been fully explored, its involvement in other cancers suggested it could also be crucial in bladder cancer development.

Hence, *KDELR2* was considered a promising therapeutic target, and the development of drugs aimed at *KDELR2* could provide new treatment options for bladder cancer patients. With our endeavors, it is anticipated that this research will offer personalized diagnostic and treatment strategies as well as immunotherapy guidance for bladder cancer patients for patients with bladder cancer to enhance prognosis and diminish mortality rates.

## 2 Materials and methods

### 2.1 Data source

The scRNA-seq data for bladder cancer, sourced from Gene Expression Omnibus (https://ww.ncbinlm.nih.gov/geo/) (GSE135337), was utilized in this study. Data pertaining to bulk RNA-seq was acquired from the Cancer Genome Atlas (TCGA) website (https://portal.gdc.cancer.gov/), which included clinical details (age, gender, ethnicity) and somatic mutation information for bladder cancer patients. By leveraging publicly accessible data, the quirement for ethical approval was bypassed.

### 2.2 Single‐cell sequencing

The gene expression data was processed in R (v4.2.0) with the Seurat package (v4.3.0). Low-quality cells were filtered out based on stringent criteria, including nFeature range (300–5000), nCount range (500–50,000), and limits on mitochondrial ≤ (1) and erythrocyte gene expression ≤ (5 contributions to the total gene count. A total of 40,167 cells were obtained after quality control.

Seurat’s NormalizeData prepared the gene expression data. The FindVariableFeatures function pinpointed the 2,000 most variable genes (6–10). ScaleData functions (v3.1.4) prepared the gene expression data (11), guiding subsequent principal component analysis. The harmony R package (v.0.1.1) (12, 13) mitigated batch effects between sample.CellCycleScoring is used to calculate cell cycle phases (14). Clusters were formed based on the top 30 principal components (PCs). For UMAP visualization, the same 30 PCs were selectively used to depict gene expression patterns (15–18). A dimensionality reduction of 30 and a resolution of 1.2 were used.

### 2.3 Identification of cell subpopulations

Cell clusters were initially identified with Seurat’s FindClusters and FindNeighbors functions (18–21). Clusters were annotated based on marker genes expression averages.

### 2.4 Trajectory analysis of TCs subpopulations

Slingshot (v2.6.0) inferred cell lineages and pseudotimes, leveraging clustering-based MSTs to define lineage architecture. Synchronized master curves and branching curve fittings were applied, with getCurves used to derive smooth trajectory curves.

### 2.5 Assessment of cell stemness

The AUCell method (22) is utilized for the identification of cells exhibiting active gene single-cell RNA-seq data underwent analysis to assess gene expression profiles. It takes gene sets as input and provides an assessment of the ‘activity’ of each gene set within every individual cell. In this particular study, it was employed to evaluate the stemness characteristics associated with different tumor cell subpopulations.

### 2.6 Enrichment analysis of cellular subpopulations

Differentially expressed genes (DEGs) were identified using FindAllMarkers, applying a Wilcoxon test with thresholds of min.pct and min.diff.pct at 0.25, and a logfc threshold of 0.25 (23). ClusterProfiler (v4.6.0) facilitated DEGs enrichment and analysis using GO (24), KEGG, and GSEA (25–27). Significant GO terms were identified with an adjusted P-value < 0.05.

### 2.7 Cell communication analysis

CellChat (v1.6.1) enables quantitative inference and analysis of scRNA-seq data to decipher cellular interactions. CellChat’s netVisual diffInteraction function assesses alterations in cell-cell communication strength, while IdentifyCommunicationPatterns determines the count of distinct communication patterns. Scatter plots, heatmaps, and various visualization techniques are used to visually analyze the signals entering and exiting each cell. The CellChatDB database (http://www.cellchat.org/) is then used to identify signaling pathways and receptor pairs associated with specific types of TCs in cancer. A P-value threshold of 0.05 was set to identify statistically significant cell-cell interactions across various cell types.

### 2.8 Scenic analysis

Using pySCENIC (v0.10.0) in Python 3.7, we inferred single-cell regulatory networks and clustered tumor cell subpopulations. GRNBoost identified TF target genes, which were then refined by DNA-motif analysis. AUCell scored regulon activities, revealing the top 5 transcription factors (TFs) with most significant changes in expression, based on human gene motif rankings from https://resources.aertslab.org/cistarget/.

### 2.9 Development and Verification of a Prognostic Prediction Model

The purpose of this research was to evaluate the prognostic potential of specific prognostic genes associated with diverse bladder cancer subpopulations in predicting patient survival outcomes. Through a rigorous process involving univariate and multivariate Cox proportional hazards analysis (28, 29), combined with Least Absolute Shrinkage and Selection Operator (LASSO) regression, we pinpointed the most influential prognostic genes as key predictors for developing a robust prognostic model. Subsequently, we formulated a risk scoring system, where the risk score is calculated as the sum of the products between individual gene expression levels and their respective coefficients.


$$\text{R}\text{i}\text{s}\text{k}\;\text{S}\text{c}\text{o}\text{r}\text{e}=\sum_{\text{i}}^{\text{n}}\text{X}\text{i}\times \text{Y}\text{i}$$

Employing an optimized cutoff threshold derived from the “surv_cutpoint” function, patients were stratified into distinct low risk and high STRS groups, allowing for a comparative analysis of prognostic differences across patient subpopulations. To visualize and statistically validate the predictive capabilities of our risk score model, we leveraged the “Survival” package (v3.3.1) in R for survival analysis and utilized the “ggsurvplot” function to generate survival curves (30–32). Moreover, we ensured the reliability of our model by assessing its accuracy and calibration through the generation of Receiver Operating Characteristic (ROC) curves (33–37) using the “timeROC” package (v0.4.0), providing a comprehensive evaluation of the model’s predictive performance.

### 2.10 Immune microenvironment analysis

We utilized the CIBERSORT R package (version 0.1.0) to compute immune-related scores for immune cells, providing a comprehensive evaluation of the patients’ immune milieu. Furthermore, we scrutinized the levels of immune cell infiltration and differential gene expression associated with immune checkpoints, and conducted investigations into the correlation between risk scores, immune cells, and model genes (38). Concurrently, we employed the Tumorlmmune Dysfunction program to appraise the response to tumor immunotherapy.

### 2.11 Identification of malignant cells by inferCNV

To distinguish between cancerous and non-cancerous cellular populations, we initially estimated the baseline copy number variation (CNV) across various genomic regions by analyzing disruptions in chromosome gene expression patterns. This was accomplished utilizing the inferCNV R package (accessible at https://github.com/broadinstitute/inferCNV/wiki), a tool specifically designed for CNV inference. Using endothelial cells (ECs) as a benchmark, we leveraged the inferCNV algorithm to characterize the CNV landscape within distinct cellular subpopulations. Subsequently, those EPCs subpopulations that exhibited marked alterations in their CNV profiles, indicative of significant genomic instability, were identified and classified as malignant cells, thereby facilitating the differentiation between cancerous and non-cancerous cell types.

### 2.12 Cell culture

The UM-UC-1 tumor-derived cell line was propagated in MEM medium under standardized conditions (37°C, 5% CO2 atmosphere, and 95% humidity) supplemented with 10% fetal bovine serum (FBS) and 1% antibiotics. Similarly, the VM-CUB1 cell line was maintained in DMEM medium under identical conditions, also enriched with 10% FBS and 1% antibiotics to ensure optimal growth and health.

### 2.13 Cell transfection

RNA constructs sourced from GenePharma (Suzhou, China) facilitated the downregulation of *KDELR2* expression. The cells were seeded onto a 6-well plate at a moderate density of 50%, subsequently subjected to transfection procedures involving *KDELR2*-specific knockdown constructs (si-*KDELR2*-1 and si-*KDELR2*-2), as well as a negative control construct (si-NC) for comparison. Lipofectamine 3000RNAiMAX (Invitrogen, USA) was used for transfection under manufacturer directions. Every si-RNA (RIbbio, China) was transfected into cells. siRNA sequences: si-1: GUAGUCCAGACCAUCCUAU; si-2: UCGUGCUUUGUAUCUUGUC. qRT-PCR Primers: F: TGGATCTGGCGCTTCTACTT; R: GCTGGCAAACTGAGCTTCTT.

### 2.14 Western blotting

After the transfected cells attained a 70% confluency level, they were lysed in RIPA buffer. The resulting lysates were clarified through centrifugation at 12,000 rpm for 15 minutes, preparing them for SDS-PAGE separation. The separated proteins were then transferred onto PVDF membranes, which were subsequently blocked with 5% BSA for 1.5 hours at ambient temperature. Following an overnight incubation with an Anti-*KDELR2* antibody at 4°C, the membranes were further incubated with a secondary antibody for an hour. Ultimately, the presence of *KDELR2* protein bands was detected using an ECL Western Blot substrate for visualization.

### 2.15 Quantitative real-time polymerase chain reaction

The RNA extraction process involved the utilization of Trizol reagent, Trizol reagent was used to lyse cells and release RNA, with chloroform and isopropanol employed to precipitate the RNA while suppressing RNase activity. Throughout the extraction process, it was essential to confirm that the workbench, tools, and water utilized were RNase-free to avoid RNA degradation. Followed by a reverse transcription step facilitated by the PrimeScript™ Kit. Subsequently, the quantitative Real-time Polymerase Chain Reaction was performed using SYBR Green premix as the fluorescent dye for amplification detection.

### 2.16 Cell viability assay

To assess the viability of UM-UC-1 and VM-CUB1 cells post-transfection, the Cell Counting Kit-8(CCK-8) assay was employed. Cells were seeded in 96-well plates at a density of 5×10³ cells per well and allowed to incubate for 24 hours. Subsequently, 10μL of CCK-8 labeling reagent (A311-01, Vazyme) was added to each well, and the plates were incubated in the dark at 37°C for two hours. From day one to day four, cell viability was quantitatively determined by measuring the absorbance at 450nm using an enzymatic marker (A33978, Thermo). The average optical density values were computed and plotted on a line graph to visually represent the cellular viability trends over time.

### 2.17 Transwell assay

Prior to the experiment, the cells underwent a 24-hour serum starvation period in medium devoid of serum. Afterward, the cell suspension was mixed with Matrigel (BD Biosciences, USA) and seeded into the upper chamber of Costar plates, while the lower chamber was filled with serum-rich medium to create a chemoattractant gradient. The cells were then incubated for 48 hours in a cell culture incubator to allow for migration and invasion. Following incubation, the cells were fixed with 4% paraformaldehyde and stained with crystal violet to visually assess their invasive potential.

### 2.18 Wound healing assay

The stably transfected cells were plated in 6-well dishes placed in the incubator and cultured at 37°C with 5% CO₂ until the cells reached confluence. Using a sterile 200μL pipette tip, uniform scratches were generated across the cell monolayer in each well. Subsequently, the wells were gently washed with PBS to remove any dislodged cells or debris. The scratched areas were then subjected to incubation in serum-free medium to monitor cell migration. Images of the scratch wounds were documented at 0 hours and again after 48 hours of incubation, with the widths of the scratches measured utilizing Image-J software for quantitative analysis.

### 2.19 5-Ethynyl-2’-deoxyuridine proliferation experiments

The UM-UC-1 and VM-CUB1 cell lines, post-transfection, were seeded at a concentration of 5×10³ cells per well in 6-well plates. Following a 24-hour incubation period at ambient temperature, the EdU working solution was introduced into the culture medium and allowed to incubate for 2 hours. Subsequently, the cells underwent a double wash with PBS and were fixed using a 4% paraformaldehyde solution for 15 minutes to stabilize them. After fixation, the cells were permeabilized and quenched with a mixture of 2 mg/ml glycine and 0.5% Triton X-100 for 15 minutes. Finally, the cells were stained with a combination of 1X Apollo solution (1 ml) and Hoechst staining reaction solution (1 ml), followed by a 30-minute incubation period. Fluorescence microscopy was then employed to assess cell proliferation by capturing images of the stained cells.

### 2.20 Statistical analysis

R and Python software packages are utilized for analyzing data from databases, while GraphPad Prism, specifically version 8.0.1, serves as the tool of choice for experimental data analysis. Throughout the analyses, two-tailed p-values are employed, and statistical significance is determined based on values falling below the threshold of 0.05.

## 3 Results

### 3.1 Single-cell sequencing analysis revealed major transcriptomic features of the TME in bladder cancer

**Figure 1:**
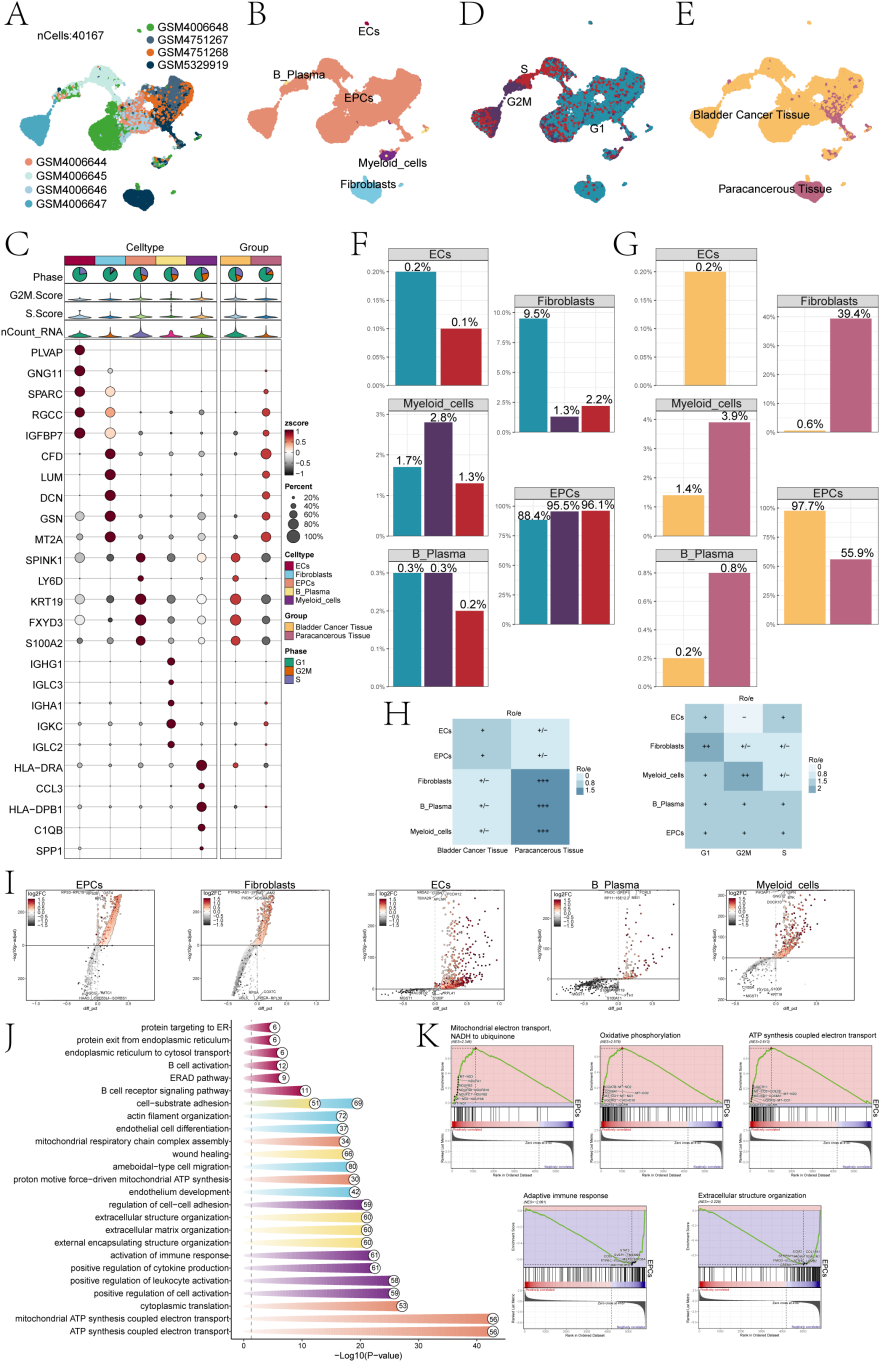
Single-cell profiling of bladder cancer identified 5 cell types. **(A)** The UMAP plot visualized the analysis encompassing all cells from eight bladder cancer samples sourced from seven patients,by using single-cell RNA sequencing method(n=40,167). **(B)** UMAP plot showed clusters of five different types of cells (ECs; EPCs; B plasma cells; myeloid cells; fibroblasts). **(C)** The bubble chart displayed the top 5 marker genes associated with each individual cell cluster. The bar charts were color-coded by cell subpopulation, and the pie charts illustrated the proportion of each phase. The violin plots visualized the expression levels of G2M.Score, S.Score, and nCount-RNA, with bubble size representing the percentage of gene expression and color indicating z-score. **(D)** UMAP plot illustrated the cellular distribution at three different cell cycle phases (Phase: G1, G2M, S). **(E)** UMAP plot showed the distribution of cell samples from bladder cancer tissue and paracancerous tissue. **(F)** The bar charts represented the proportions of three cell phases in five different types of cells. **(G)** The bar charts represented the proportions of bladder cancer tissue and paracancerous tissue in 5 different types of cells. **(H)** The heatmap revealed the distribution preferences of different cell subpopulations in terms of sample origin and cell cycle phase. **(I)** Volcano plots showed different expressed genes in 5 types of cells. **(J)** Enrichment analyses of DEGs across all cells unveiled their key biological roles and functions. **(K)** GSEA identified both positively and negatively enriched biological pathways in EPCs, including mitochondrial electron transport, NADH-ubiquinone reduction, oxidative phosphorylation coupled ATP synthesis, adaptive immune response, and extracellular structure organization.

Based on our research objectives and requirements, we conducted a comprehensive review of the scRNA-seq data related to bladder cancer in public database. To explore the cellular transcriptome characteristics of the bladder cancer TME, we performed a systematic and comprehensive analysis of the collected scRNA-seq data. The dataset was obtained from seven primary tumor tissue samples and one paracancerous sample from seven bladder cancer patients that included diverse demographic backgrounds and clinical stages of bladder cancer. This diversity enhanced the relevance of our findings to a broader patient population. After removing batch effects and primary quality control, we obtained 40,167 high-quality cells (**Figure 1A**). Dimension reduction cluster analysis was performed on the screened cells, and we found that they could be divided into 5 cell types, including B plasma cells, ECs, epithelial cells (EPCs), myeloid cells, and fibroblasts (**Figure 1B**). The bubble plot depicted the levels of expression for the most significant genes in distinct cellular subpopulations, categorized by cell types and tissue samples (**Figure 1C**). The results found that ECs highly expressed *PLVAP, GNG11, SPARC, RGCC, IGFBP7*; fibroblasts highly expressed *CFD, LUM, DCN, GSN, MT2A*; EPCs showed high expression of *SPINK1, KRT19, LY6D, FXYD3* and *S100A2;* B plasma cells showed high expression of *IGHG1, IGLC3, IGHA1, IGKC, IGLC2*; myeloid cells showed high expression of *HLA-DRA, CCL3, HLA-DPB1, C1QB* and *SPP1*; bladder cancer tissue cells showed high expression of *SPINK1, LY6D, KRT19, FXYD3, S100A2;* paracancerous cells highly expressed genes including *CFD, LUM, DCN, GSN, MT2A,RGCC* and so on. In conclusion, we could find that EPCs marker genes had high consistency with the highly expressed genes in cancer tissues. At the same time, we could observe that the cell phases are different in these 5 cell types, and most of the cells were in G1 phase (**Figure 1D**). Moreover, most ECs and EPCs were mainly derived from bladder cancer tissue, while fibroblasts were mainly derived from paracancerous cells (**Figure 1E**). The above study were consistent with Ro/e analysis, that EPCs tended to be derived from bladder cancer tissues (**Figure 1H**). Through cell cycle analysis of cell subpopulations, each cell subpopulation tended to be distributed in different cycle phases (**Figures 1C, D, F**). In addition, the proportion of EPCs in bladder cancer tissue cells and paracancerous cells was much greater than that of other cells, especially in bladder cancer tissue cells, as high as 97.7% (**Figures 1E, G**).

Volcano plots results showed that the different genes among ECs, fibroblasts, EPCs, B plasma cells, and myeloid cells (**Figure 1I**). To investigate the biological processes of different cell types, by using GO-BP enrichment analysis (**Figure 1J**), we identified that EPCs were mainly enriched in ATP synthesis coupled electron transport, mitochondrial ATP synthesis coupled electron transport, cytoplasmic translation, proton motive force-driven mitochondrial ATP synthesis and mitochondrial respiratory chain complex assembly. Further GSEA (**Figure 1K**) of EPCs was performed according to GO-BP terms. The results showed that pathways such as mitochondrial electron transport, NADH to ubiquinone; oxidative phosphorylation and ATP synthesis coupled electron transport showed a positive enrichment trend, by comparison, pathways such as adaptive immune response and extracellular structure organization showed a trend of negative enrichment in this genome.

### 3.2 Visualization analysis of bladder cancer tissue cells

**Figure 2:**
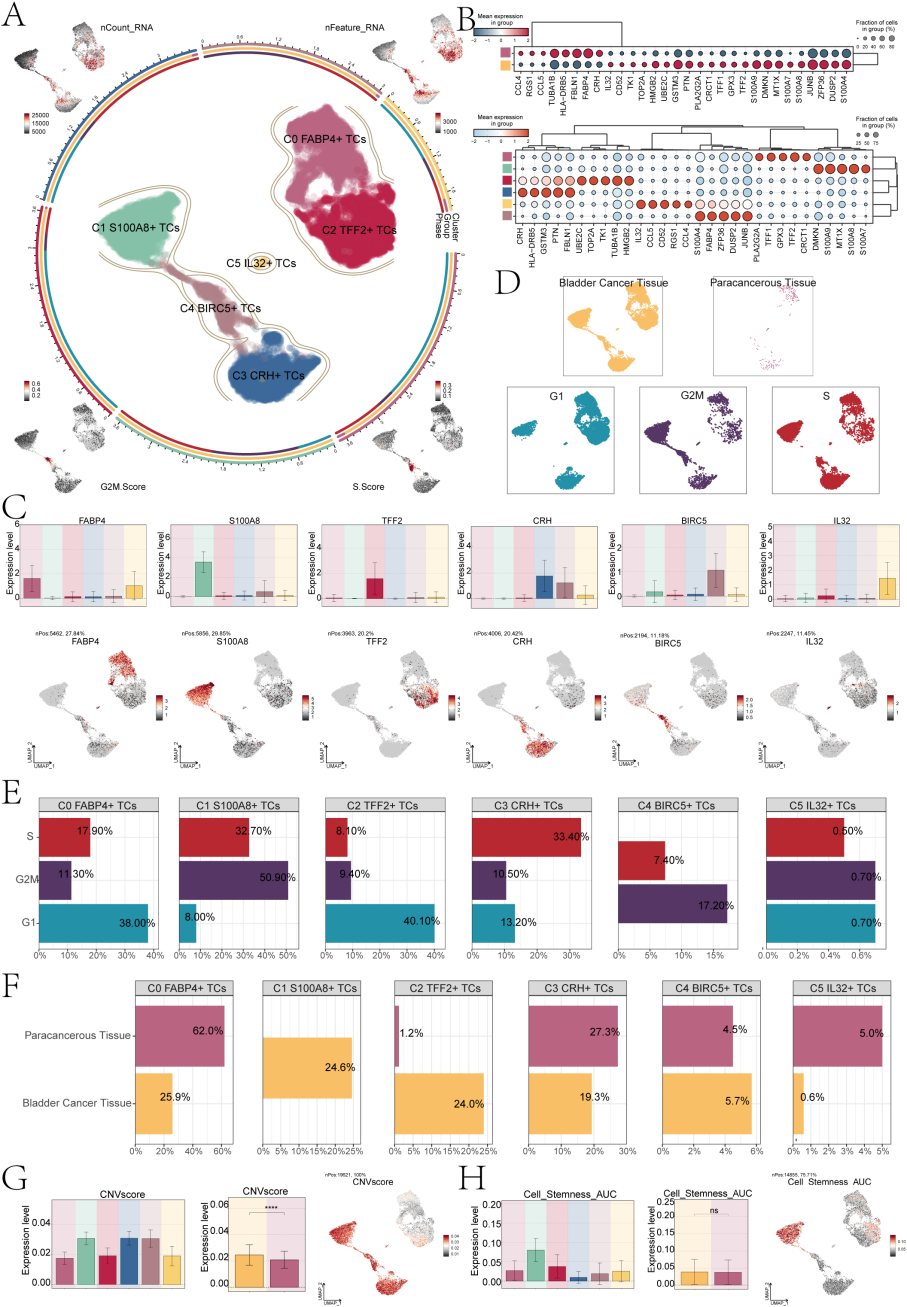
*S100A8*+ TCs specifically expressed in malignant EPCs and are associated with cell stemness. **(A)** The circle plot represented the clustering of the six tumor cell subpopulations identified in bladder cancer. and the contour curve outlines the boundaries of each cell subpopulation. The outer axis of the circle plot represents the logarithmic scale of the entire cell count in each cell category. The three-color tracks representative the ratio of each tumor cell subpopulation in cell types, cell sample types, and cell phases, respectively, and are colored according to cell categories. The UMAP graphs in the four corners start from the upper left corner and go clockwise to show the expression distribution of nCount-RNA, nFeature-RNA, S.score, and G2M.score across all TCs was shown. **(B)** The bubble charts showed the manifestation of marker genes in two sample tissues (top) and in six tumor cell clusters (bottom). **(C)** The bar charts and UMAP plots collectively presented the expression profiles of six marker genes *FABP4*, *S100A8*, *TFF2*, *CRH*, *BIRC5*, and *IL32* across six tumor cell clusters. **(D)** UMAP visualized the distribution of TCs in bladder cancer tissue, paracancerous tissue, and G1, G2M, and S phases. **(E)** The bar charts showed the percentage of G1, G2M, and S phases in six tumor cell clusters. **(F)** The bar charts illustrated the percentage of bladder cancer tissue and paracancerous tissue across six tumor cell clusters. **(G)** The bar plots illustrated the CNV score expression levels across six tumor cell clusters, bladder cancer tissue, and paracancerous tissue. Meanwhile, the UMAP plot visualized the distribution pattern of CNV scores. ****P < 0.0001. **(H)** The bar plots showed the AUC score of cell stemness for six tumor cell clusters and bladder cancer tissue and paracancerous tissue. The UMAP plot showed the distribution of cell stemness AUC score. "ns" was used to say that there was no significant difference.

The findings of various studies have demonstrated that the presence of TCs can trigger significant alterations at a molecular, cellular, and physical level within the surrounding tissue, leading to the formation of a specialized environment known as the TME. During the initial stages of tumor development, there is an intricate interplay between cancer cells and various components of this microenvironment, facilitating tumor survival, local infiltration, and metastatic dissemination (39). Therefore, TCs are the real culprit of tumor development. We first used the infer CNV algorithm to successfully separate 19,621 bladder cancer cells from EPCs based on CNV (**Supplementary Figure S1**). Next, we identified six subpopulations of bladder cancer cells based on the level of marker genes expression. The circle diagram showed six cell subpopulations. These were C0 *FABP4*+ TCs, C1 *S100A8*+ TCs, C2 *TFF2*+ TCs, C3 *CRH*+ TCs, C4 *BIRC5*+ TCs and C5 *IL32*+ TCs. In addition, we used UMAP plots to show the nCount-RNA, nFeature-RNA, G2M.Score and S.Score of each subpopulation (**Figure 2A**). Subsequently, the bubble plots showed the marker genes of different tissue cells and subpopulations (**Figure 2B**). According to the comparison of named gene expression of each subpopulation of tumor cell in bar charts, C0 was characterized by high expression of *FABP4*. The high expression of *S100A8* characterizes C1, while C2 was characterized by the elevated expression of *TFF2*. Similarly, C3 was distinguished by its heightened expression of *CRH*, and C4 exhibits a notable increase in *BIRC5* expression. Lastly, the high expression of *IL32* served as a defining feature for C5. UMAP plots showed the named gene expression of each subpopulation, which was consistent with the results of bar charts (**Figure 2C**).

For further analysis of the subpopulations of TCs, we conducted deeper visualization study. First, we performed sample sources and cell cycle phases analysis of TCs, and combined the UMAP plots to observe the main distribution of different TCs (**Figure 2D**). The results showed that most of the C1 subpopulation of TCs in G2M stage, compared to the other subpopulations, the highest proportion, as high as 50.90%,at the same time, the tissue classification results show that the C1 subpopulation is different from other subpopulations, its mainly come from bladder cancer tissue cells (**Figures 2E, F**), and the CNV score was relatively high (**Figure 2G**). In addition, we conducted CNV score analysis of bladder cancer tissue cells and paracancerous cells, the cancer tissue cell expression level of CNV score was higher than the paracancerous cells. There were significant differences between them. UMAP plot showed the CNV score distribution characteristics. Additional examination of the stemness characteristics of specific subpopulations of cells in bladder cancer demonstrated that the C1 subpopulation had the highest Area Under the Curve (AUC) score for stemness. This meant that C1 had the characteristics of low differentiation degree and strong differentiation potential. The stemness score of bladder cancer cells exhibited a slightly higher level compared to that of paracancerous cells; however, the difference did not reach statistical significance (**Figure 2H**).

### 3.3 Heterogeneity of stemness and development in bladder cancer cell subpopulations

**Figure 3:**
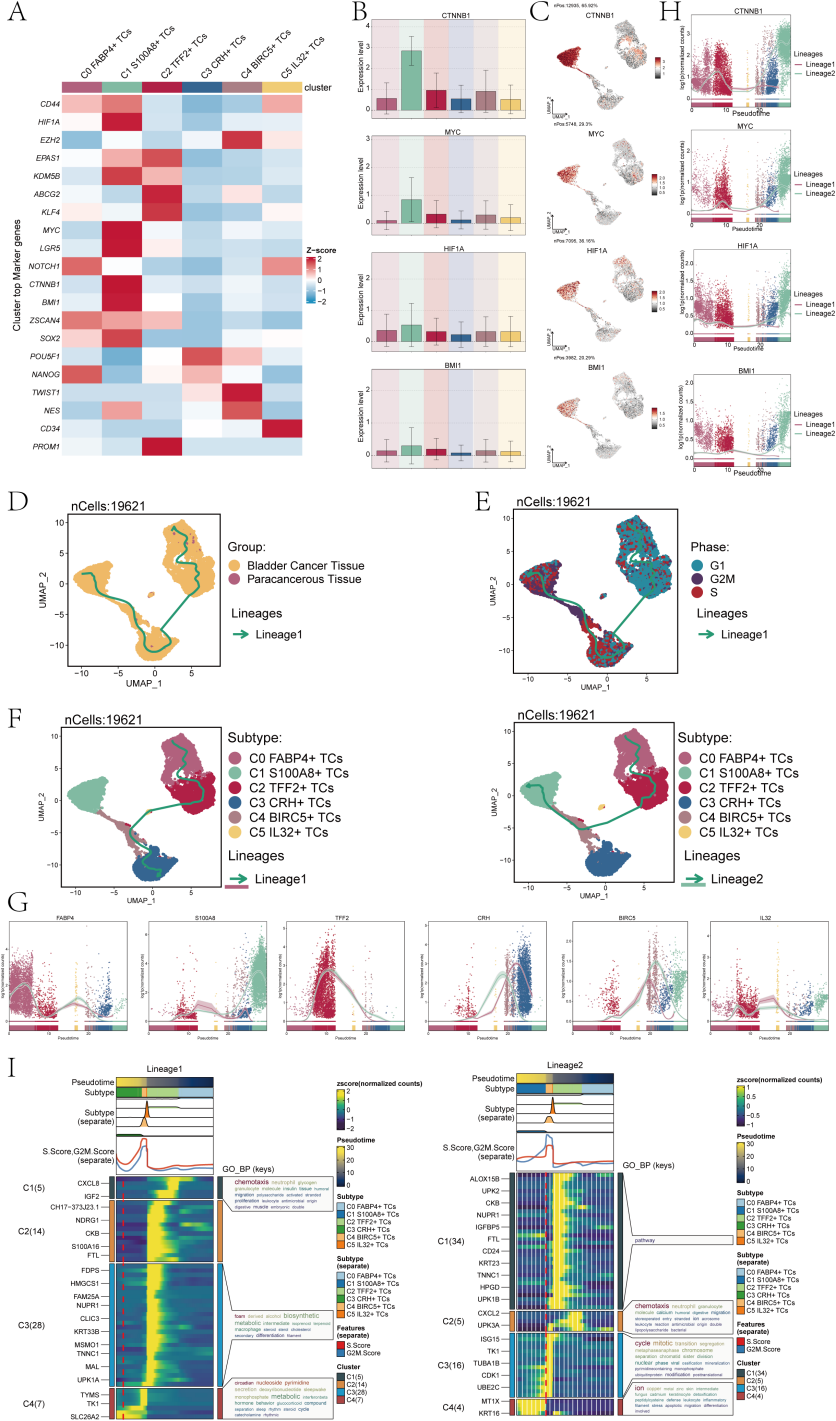
Analysis of tumor cell clusters for cell pluripotency and analysis of the developmental trajectory of cells. **(A)** The heatmap showed the z-scores of the marker genes related to cellular stemness in six cell clusters. **(B)** The bar plots displayed the expression levels of four key genes associated with cell stemness across six cellular clusters. **(C)** The UMAP plots showed the dispersion of the four genes related to cellular stemness within all TCs. **(D)** The UMAP plot illustrated the tumor cellular trajectory changes inferred based on bladder cancer tissue and paracancerous tissue. **(E)** The UMAP plot illustrated the temporal trajectories of tumor cellular differentiation, depicted based on three cell cycle phases: G1, G2M, and S. The lineage showed the trajectory from G1 and S to G2M. **(F)** The UMAP plots showed the two lineages of cellular differentiation over time for the six tumor cell clusters discussed. The lineage showed the trajectory from C0 *FABP4*+ TCs to C3 *CRH*+ TCs(left), another lineage showed the trajectory from C0 *FABP4*+ TCs to C1 *S100A8*+ TCs (right). **(G)** The dynamic trend graphs showed the expression of six marker genes over time at different differentiation stages. **(H)** The dynamic trend graphs showed the expression of four stemness genes over time at different stages. **(I)** The heatmaps showed GO enrichment pathways during the differentiation process of TCs. The top bar chart represents pseudo-time and six different types of cells. The faceted mountain plot showed the distribution density of six tumor cell subpopulations spanning various pseudo-time stages. The trajectory plot showed the expression of S.Score and G2M. Score (red represented S.Score, blue represented G2M. Score) as they changed with pseudotime.

Subsequently, we conducted an analysis of gene expression related to cell stemness in the subpopulations of TCs. And we found that the stemness gene expression level was more significant in the C1 subpopulation compared with the other cell subpopulations (**Figure 3A**). The bar charts confirmed the above conclusion that the C1 subpopulation is higher in *CTNNB1, MYC, HIF1A* and *BMI1* expression levels than other subpopulations cells (**Figure 3B**). Research had indicated that the atypical activation of *CTNNB1* was linked to the development of various types of tumors, including but not limited to colorectal cancer, ovarian cancer, prostate cancer, hepatoblastoma, and hepatocellular carcinoma (40). *HIF1A*, hypoxia-inducing factor, promotes angiogenesis and is important for the vascular system in the embryo and for cancer tumors (41, 42). As a proto-oncogene, *BMI1* was an important component of the polycomb gene family. High levels of *BMI1* expression have been found to be significantly associated with the onset, progression, and prognosis of diverse malignancies. *BMI1* could participate in tumorigenesis by inhibiting multiple gene loci, synergizing with other proto-oncogenes, and enhancing telomerase activity (43). The synergistic effect of these genes precisely demonstrateed the pro-tumor effect of C1 subpopulations. The UMAP plots showed the expression distribution of *CTNNB1, MYC, HIF1A, BMI1* genes (**Figure 3C**).

Cell stemness was closely related to cell differentiation and development. To explore the differentiation trajectory of tumor cell subpopulations, we performed a pseudotime analysis and presented it using UMAP plots. First, we showed the lineage trajectory of bladder cancer tissue cells and paracancerous cells (**Figure 3D**), and the cell cycle lineage trajectory (**Figure 3E**). The initial cell differentiation trajectory began in the G1 phase and gradually transitioned to the S and G2M phases according to the cell cycle sequence. We then analyzed six different TCs subpopulations, and the UMAP plots showed two main cell lineage tracks (**Figure 3F**), including lineage 1:C0→C2→C5→C4→C3; lineage 2:C0→C2→C4→C1. The difference between the two trajectories mainly existed in the later stage. Combined with **Figure 3D**, we could find that the ended of lineage 1 ended mainly C3 subpopulation, which contained cells from both tumor tissues and cells from paracancerous cells. The end of lineage 2 ended mainly C1 subpopulation, which was a tumor cell subpopulation derived entirely from a bladder cancer tissue sample. Combined with the evolution of TCs and based on the tissue origin, CNV score and cell stemness of C1 subpopulation, the conclusion that C1 subpopulation has a high degree of malignancy and is closely related to the progression of bladder cancer was reaffirmed.

Furthermore, we conducted an analysis on the temporal expression patterns of marker genes belonging to 6 distinct subpopulations. The findings indicated that. different from other cell subpopulations. The high expression of marker genes for the C1 subpopulation was predominantly observed during the later stage (**Figure 3G**). To confirm the above findings, we also conducted a temporal analysis of the expression of cell stemness genes. The primary distinction between lineage1 and lineage2 was observed in the later stage, with a notable increase in stemness gene expression detected in lineage2 compared to lineage1 (**Figure 3H**). This was consistent with previous conclusions. Subsequently, we analyzed the subpopulations using GO-BP enrichment analysis to verify the related biological processes of the two lineages (**Figure 3I**), and the dynamic timing showed the expression changes of the different genes of TCs along the two trajectories within the pseudotiming.

### 3.4 Heterogeneity of biological functions of bladder cancer TCs

**Figure 4:**
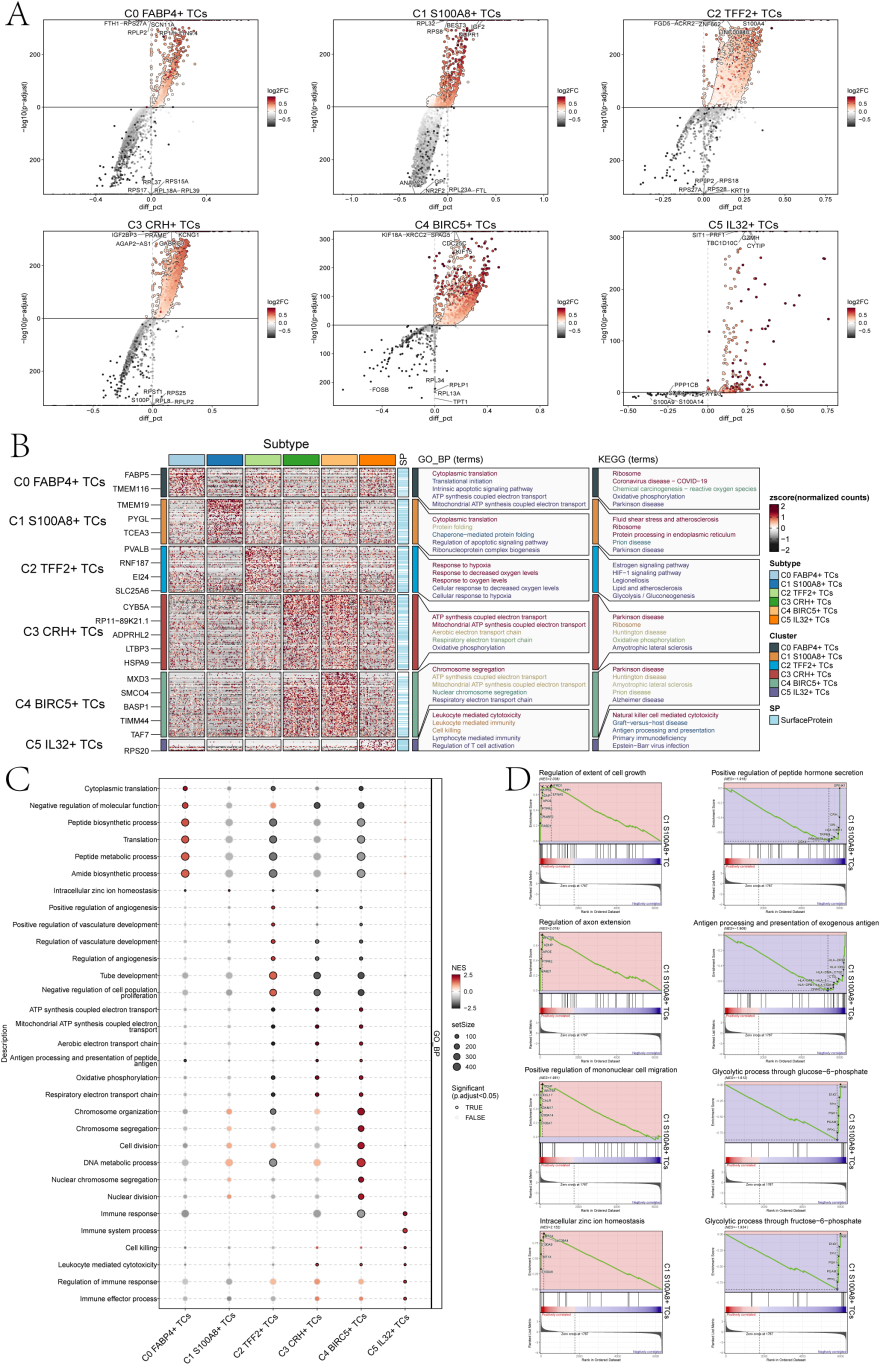
Perform gene and pathway enrichment analysis for each tumor cluster set. **(A)** The volcano plots showed the differential gene expression signatures across the six clusters. **(B)** The heatmap displayed the top five enrichment pathways among the six clusters identified through GO-BP and KEGG enrichment analysis. **(C)** The bubble plot showed the GSEA results of the six tumor cell clusters. **(D)** GSEA analyzed eight positively or negatively enriched pathways in C1 *S100A8*+ TCs.

To explore the biological functions of TCs in bladder cancer, we first analyzed the different expression genes of six tumor cell subpopulations. The results showed that the main upregulated genes in C1 *S100A8*+ TCs were *RPL32, RPS8, BEST3, IGF2* and *NUPR1* (**Figure 4A**). Among them, *RPL32* played a crucial role in modulating cellular signaling pathways. It had found that *RPL32* could participate in multiple signaling pathways during tumorigenesis and significantly contribute to the initiation and progression of neoplastic growth (44, 45). *RPS8* was mainly involved in protein folding and stability, and in tumor studies, *RPS8* has the potential to serve as a biomarker specific to tumors due to its tendency for elevated expression levels in tumor tissues and cells compared to normal tissues (46). *BEST3* was a coding gene that encoded proteins with good histocompatibility and low expression levels. *BEST3* had been observed to be present in multiple types of cancerous tissues, and its expression level appears to be linked with the tumor’s capacity for invasion. Furthermore, the correlation between the expression level of *BEST3* and the efficacy of tumor therapy has prompted its investigation as a potential therapeutic target or biomarker (47). The role of *IGF2* was pivotal in regulating cellular proliferation, development, motility, differentiation, and viability. Furthermore, *IGF2* played a role in a variety of cancer development (48). *NUPR1* had been linked to the onset and progression of cancer, and it was commonly observed to be upregulated in different cancer forms. Moreover, *NUPR1* could act as a transcriptional regulator affecting the expression of genes involved in cell cycle regulation, apoptosis, and stress response. These genes had a significant correlation with the onset and progression of neoplasms (49, 50).

Furthermore, we conducted enrichment analysis using GO-BP and KEGG to identify enriched biological processes and pathways associated with the DEGs in the subpopulations of TCs. And the heatmap showed the results of the genes enrichment in the TCs subpopulations (**Figure 4B**). C0 *FABP4*+ TCs was mainly associated with cytoplasmic translation, translational initiation, intrinsic apoptotic signaling pathway, ATP synthesis coupled electron transport and mitochondrial ATP synthesis coupled electron transport biological processes related in GO-BP, and was associated with the ribosome, coronavirus disease-COVID-19,chemical carcinogenesis-reactive oxygen species, oxidative phosphorylation and parkinson disease pathways in KEGG.C1 *S100A8*+ TCs was mainly associated with cytoplasmic translation, protein folding, chaperone-mediated protein folding, regulation of apoptotic signaling pathway and the biological process of ribonucleoprotein complex biogenesis in GO-BP, and was associated with the fluid shear stress and atherosclerosis, ribosome, protein processing in endoplasmic reticulum, prion disease and parkinson disease pathways in KEGG. The association of C2 *TFF2*+ TCs primarily pertained to the biological processes related to hypoxia response, decreased oxygen levels response, oxygen levels response, cellular response to decreased oxygen levels, and cellular response to hypoxia in GO-BP, and was associated with the estrogen signaling pathway, HIF-1signaling pathway, legionellosis, lipid and atherosclerosis and the glycolysis pathways correlation in KEGG. The association of C3 *CRH*+ TCs primarily pertained to the process of ATP production through electron transport, mitochondrial ATP synthesis coupled with electron transport, aerobic electron transport chain, and oxidative phosphorylation in GO-BP, and was associated with the parkinson disease, ribosome, huntington disease, oxidative phosphorylation and the amyotrophic lateral sclerosis pathways correlation in KEGG.C4 *BIRC5*+ TCs was mainly associated with chromosome segregation, ATP synthesis coupled electron transport, mitochondrial ATP synthesis coupled electron transport, nuclear chromosome segregation, respiratory electron transport chain in GO-BP, and was associated with the parkinson disease, huntington disease, amyotrophic lateral sclerosis, prion disease and alzheimer disease pathways correlation in KEGG. C5 *IL32*+ TCs was mainly associated with leukocyte mediated cytotoxicity, leukocyte mediated immunity, cell killing, lymphocyte mediated immunity and regulation of T cell activation biological processes in GO-BP, and was associated with the natural killer cell mediated cytotoxicity, graft-versus-host disease, antigen processing and presentation, primary immunodeficiency and the Epstein-Barr virus infection pathways in KEGG.

In addition, we performed a further step GSEA of the six tumor cell subpopulations, and we could observe that C1 *S100A8*+ TCs were mainly enriched in intracellular zinc ion homeostasis (**Figure 4C**). GSEA results (**Figure 4D**) also showed that the C1 subpopulation was positively enriched in the regulation of extent of cell growth, regulation of axon extension, positive regulation of mononuclear cell migration, the intracellular zinc ion homeostasis, while it was negatively regulated in positive regulation of peptide hormone secretion, antigen processing and presentation of exogenous antigen, glycolytic process through glucose-6-phosphate and glycolytic process through fructose-6-phosphate.

In summary, C1 subpopulation was mainly associated with biological pathways such as protein folding and ribosomes, which promoted and accelerated tumor cell migration and invasion. Cells were very sensitive to physiological conditions, and when the physiological conditions changed, the cells would change accordingly. When the loaded proteins exceed the folding capacity of the endoplasmic reticulum stress (ERS), ERS will be caused. The core of ERS lied in the occurrence of protein misfolding, which significantly influenced the growth and viability of cancerous cells and may facilitate the progression of bladder carcinoma (51, 52). Ribosomes, as a kind of nutrient, can escort the smooth and rapid translation of viruses and provide conditions for tumor proliferation, thus affecting the progression and development of bladder cancer (53).

### 3.5 Analysis of the cell interactions in the TME

**Figure 5:**
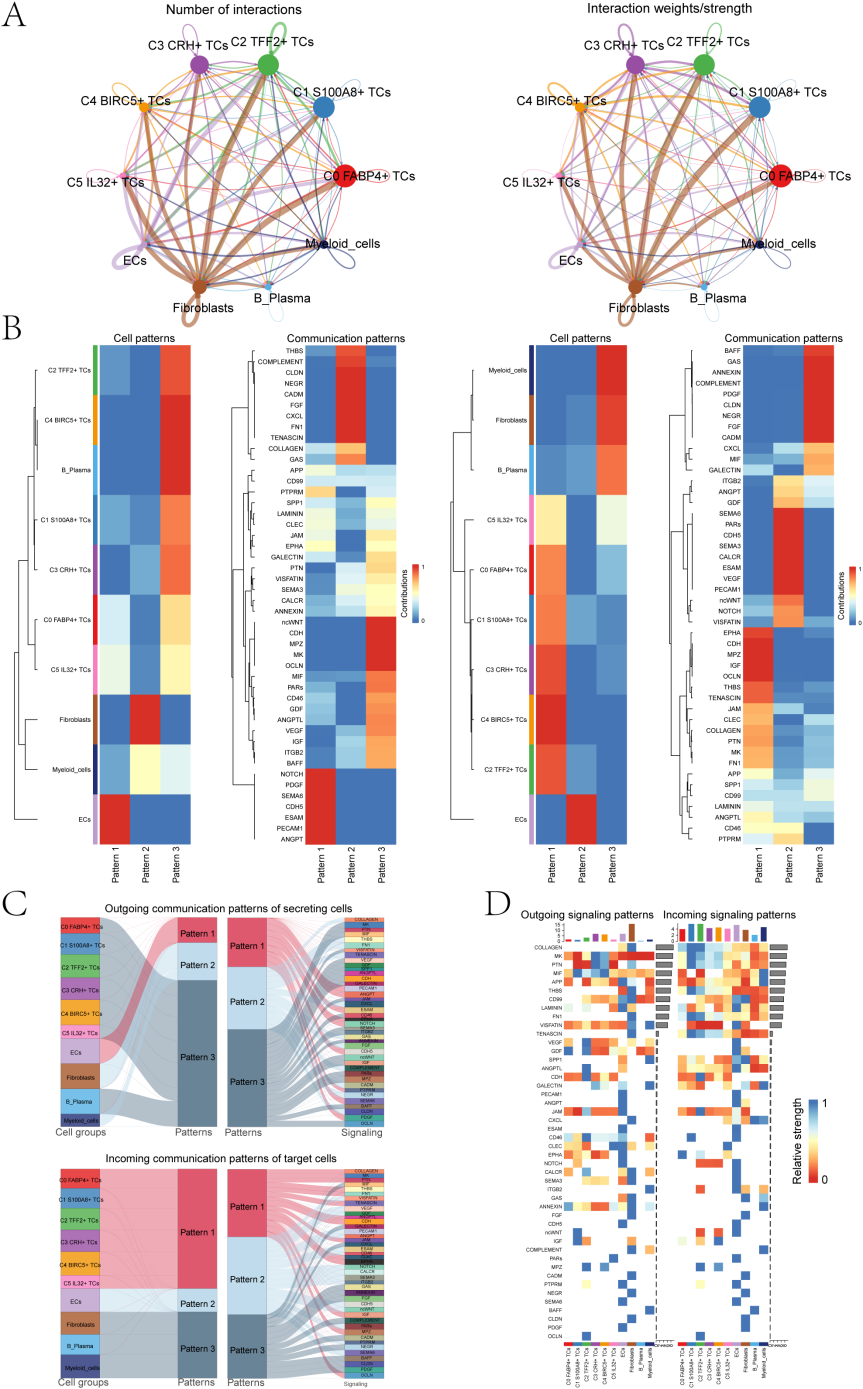
Bladder cancer cells was characterized by cell-to-cell signaling networks. **(A)** The circle charts summarized the quantity and intensity of interactions between six tumor cell clusters and four distinct cell types, providing insights into their interconnectedness. **(B)** The heatmaps separately showed the contributions of the six tumor cell clusters and four cell types in the outgoing (left) and incoming (right) signaling under three cell communication patterns, as well as the contributions of various proteins in the three communication patterns. **(C)** The Sankey diagrams illuminated the outbound communication pattern of secretory cells and the inbound communication pattern received by target cells. **(D)** The bar charts compared the relative signaling strengths of six tumor cell clusters and four cell types in both incoming and outgoing patterns. Complementarily, the heatmaps visualized the reception intensities of various proteins within these communication patterns across the same cell groups.

To gain a thorough and organized comprehension of intricate cellular reactions, our aim was to conduct an examination on intercellular connections and networks involved in ligand-receptor communication, with the intention of visualizing interactions between cells. By means of CellChat analysis, we successfully established a network for cellular communication encompassing the majority of cells, including ECs, fibroblasts, B plasma cells, myeloid cells and six tumor cell subpopulations (**Figure 5A**). Then, we determined the quantity of interactions through the ‘line’ connection linking two cell types, with thicker lines indicating a higher number of interaction paths. Additionally, we represented the strength of interactions using line weight, where thicker lines indicated stronger interactions. Among different cell subpopulations. Next, we combined the CellChat maps to explore how different cell subpopulations interact with each other through these pathways. First, we categorized cell communication patterns into three main types. In addition, the displayed heatmaps illustrated the expression of cell interaction proteins in the three modes. The two heatmaps on the left showed the outgoing signal patterns of different cell subpopulations and the active interacting proteins in the signal patterns, and the two heatmaps on the right showed the incoming signal patterns and interacting proteins of cell subpopulations (**Figure 5B**). We could observe that in the outgoing signal pattern diagrams, C1 *S100A8*+ TCs were mainly in pattern 3. Correspondingly, ncWNT, CDH, MPZ, MK, and OCLN showed high expression levels in pattern 3. Additionally, the incoming signal patterns of C1 *S100A8*+ TCs exhibited pattern 1 including but not limited to the pathways represented by such as EPHA, CDH, MPZ, IGF, and OCLN. The Sankey diagrams and heatmaps (**Figure 5C, D**) showed the communication pattern and target signal of each cell subpopulations, which was consistent with the results in **Figures 5B**.

### 3.6 Explored the cell signaling pathways in C1*S100A8*+ TCs

**Figure 6:**
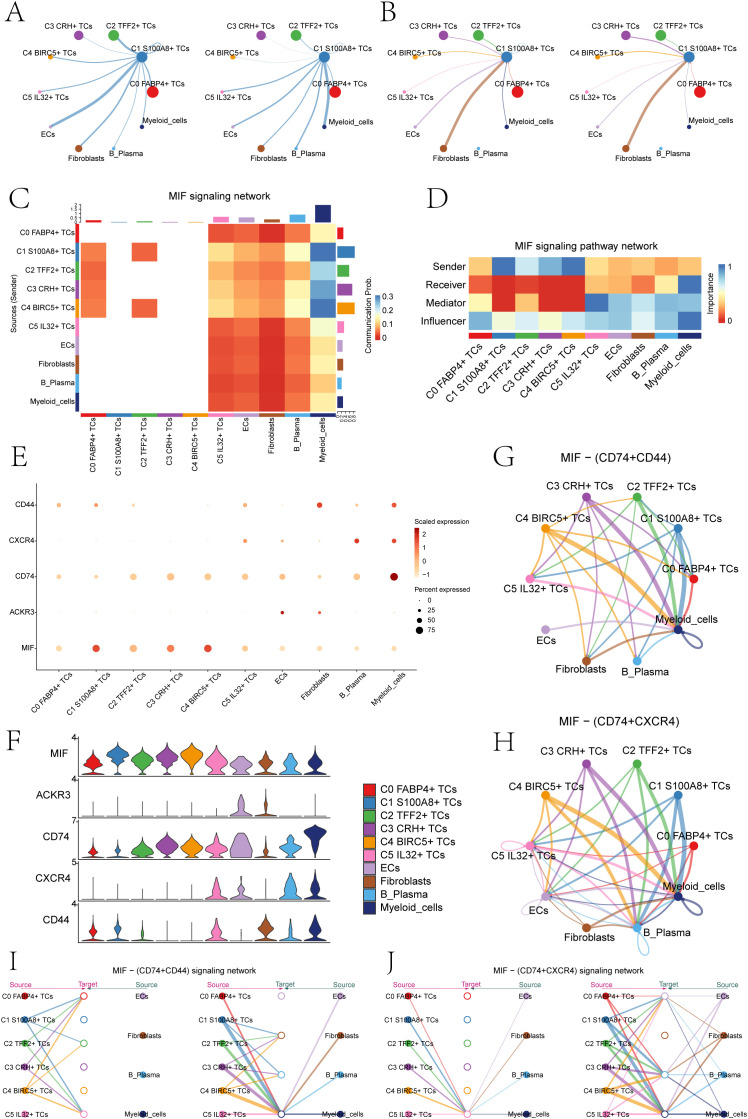
The MIF signaling network was the main communication method between *S100A8*+ TCs and Myeloid cells. **(A)** The circle diagrams showed TCs as the signal emitter and other cells as signal receiver The left side represented the communication strength, while the right side represented the number of communications. **(B)** The circle diagrams showed other cells as the signal emitter and TCs as signal receiver. The left side represented the communication strength, while the right side represented the number of communications. **(C)** The heatmap showed the communication probability of various cell clusters based on the MIF signaling pathway. There was a high probability of communication between C1 *S100A8*+ TCs and Myeloid cells. **(D)** The heatmap showed that C1 *S100A8*+ TCs mainly acted as signal senders, while myeloid cells mainly played a signal receiver and influencer in the MIF pathway. **(E, F)** The bubble chart and violin plots displayed that in the MIF pathway, the communication crosstalk between C1 *S100A8+* TCs and myeloid cells through the MIF- (CD74+CD44) and MIF-(CD74+CXCR4) ligand receptor pair. **(G, H)** The circle diagrams showed the interactions between C1 *S100A8*+ TCs and myeloid cells in the MIF- (CD74+CD44) and MIF- (CD74+ CXCR4) signaling pathways. **(I, J)** The hierarchy diagrams illustrated the autocrine and paracrine interactions between the six tumor cell clusters and ECs, fibroblasts, B plasma cells, and myeloid cells on the MIF- (CD74+CD44) and MIF- (CD74+ CXCR4) signaling pathway.

Subsequently, we analyzed the number of cell interaction strength between the C1 *S100A8*+ TCs and other cell subsets in depth (**Figures 6A, B**). We could find that both in the incoming signal or the outgoing signal, the number and intensity of the interaction between myeloid cells and C1 *S100A8*+ TCs were more significant than other cells, so we could infer that C1 *S100A8*+ TCs cells and myeloid cells had a strong interaction. Subsequently, we performed signaling network analysis and showed that C1 *S100A8*+ TCs and myeloid cells mainly communicate with MIF signals pathway (**Figure 6C**). Then we analyzed the role of the two in the signaling pathway (**Figure 6D**), which showed that C1 *S100A8*+ TCs cells mainly played the role of signal sender, while myeloid cells mainly played the role of signal sender and influencer.

By comparison, we could infer that the MIF ligand of C1 *S100A8*+ TCs cells acted on the CD74-CXCR4 and CD74-CD44 receptor of myeloid cells (**Figures 6E, F**). The cell interaction circle diagrams (**Figures 6G, H**) showed the interaction relationship between C1 *S100A8*+ TCs cells and myeloid cells in MIF-(CD74+CXCR4) and MIF-(CD74+CD44) cell signaling pathway, verifying the above statement. In order to make the results more visual, we used the interaction hierarchy diagrams (**Figures 6I, J**) to show the relationship between C1 *S100A8*+ TCs cells and myeloid cells (the thickness of the line represents the interaction strength, the thicker of the line, the relationship is more significant). The findings indicated that the intercellular communication between C1 *S100A8*+ TCs and myeloid cells primarily took place through paracrine, leading to signal crosstalk. This could disrupt normal signal transduction, leading to alterations in the tumor immune microenvironment, thereby facilitating the proliferation, metastasis, and invasion of TCs (54).

### 3.7 TFs regulate the oncogenic mechanism of C1 *S100A8*+ TCs

**Figure 7:**
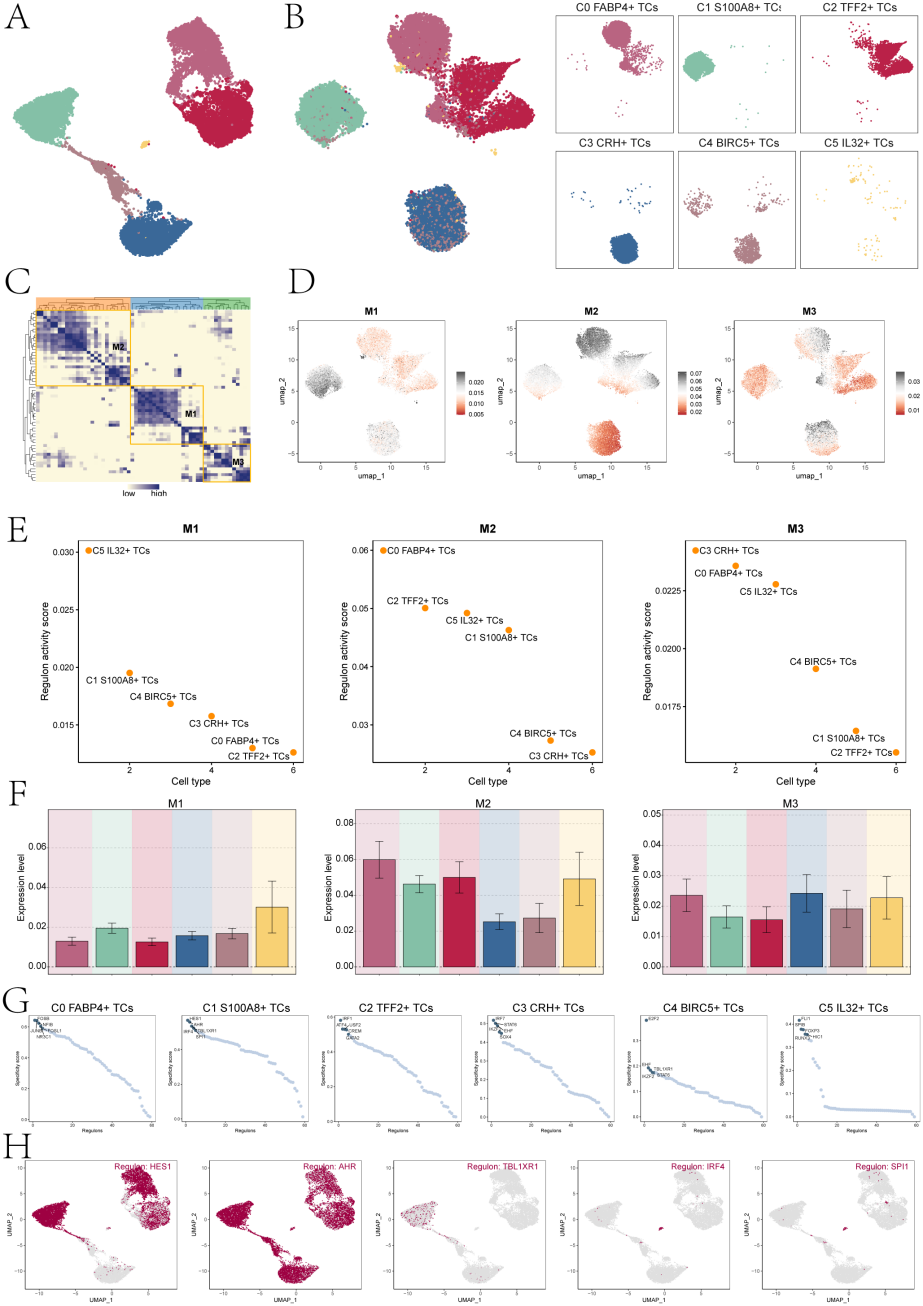
Cluster analysis of TFs and the top five TFs in C1 *S100A8*+ TCs. **(A)** The UMAP plot displayed tumor cell clustering based on gene expression levels. **(B)** The UMAP plots visualizations highlighted distinct clustering patterns among TCs, grouped according to the activation levels of various TFs. **(C)** The heatmap displayed three modules M1, M2, and M3 of transcription factor hierarchical clustering. **(D)** The UMAP plots depicted the distinct expression patterns of TFs across the three tumor cell modules. **(E)** The dot plots displayed the ranking of transcription factor regulatory activity scores for different tumor cell clusters in three modules. **(F)** The bar charts showed the expression levels of six cell clusters in three modules. **(G)** Ranking of the top 5 transcription factor activity scores of different cell types. **(H)** The UMAP plots displayed the expression of the top five TFs in C1 *S100A8*+ TCs.

TFs act on genes to regulate gene transcription by binding specific nucleotide sequences upstream of the gene, thereby affecting cell biological functions. Firstly, we conducted cluster analysis of bladder cancer tissue cells according to genes expression (**Figure 7A**). In addition, the UMAP plots displayed the expression of TFs in six tumor cell clusters (**Figure 7B**). Subsequently, based on the heatmap of TFs correlation (**Figure 7C**), we divided the TFs with similar functional and expression into three main modules, namely M1, M2 and M3. Next, we visualized the analysis of the divided modules, and observed the expression levels of each TCs subpopulation in different modules through the UMAP plots. The results showed that C1 *S100A8*+ TCs cells had significant expression in the M1 module (**Figure 7D**). To further verify the above statement, we performed transcription factor regulatory activity score analysis (**Figure 7E**), and we can observe that in the M1 module, C5 *IL32*+ TCs cells have the highest transcription factor regulatory activity score, followed by C1 *S100A8*+ TCs cell. The bar charts also verify the above statement that C1 *S100A8*+ TCs cells have high expression levels in the M1 module, second only to C5 *IL32*+ TCs cells (**Figure 7F**). Subsequently, we conducted an analysis on the top 5 TFs in the subpopulations of TCs (**Figure 7G**). The specificity score identified HES1, AHR, TBL1XR1, IRF4, and SPI1 as the top 5 TFs in C1 *S100A8*+ TCs. Finally, we used the UMAP plots to show the TFs distribution characteristics of C1 *S100A8*+ TCs cells (**Figure 7H**).

### 3.8 Constructed the prognostic model of bladder cancer

**Figure 8:**
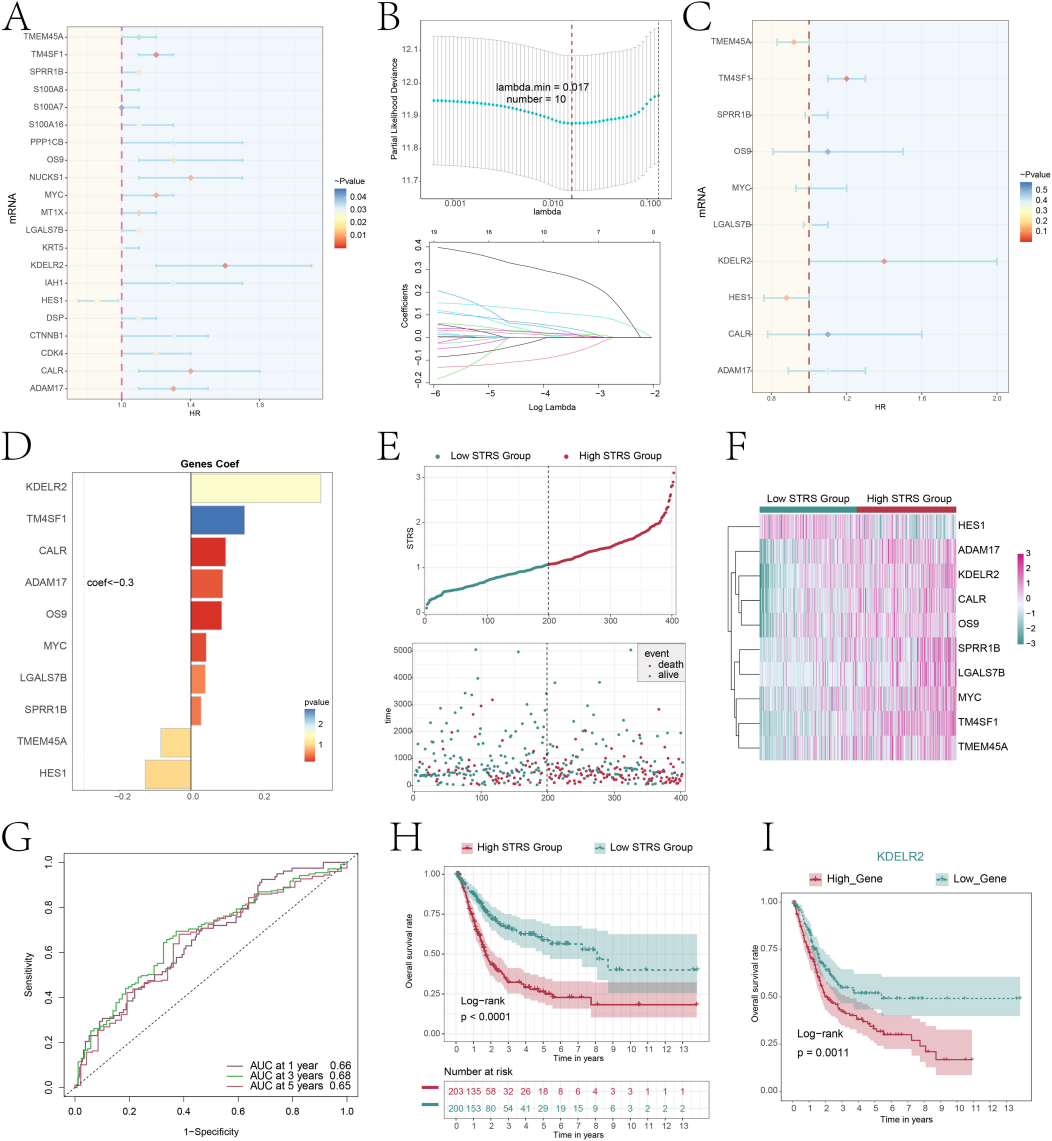
Constructing a risk prediction model through a combined approach of univariate Cox proportional hazards analysis and Lasso regression. **(A)** The forest plot displayed the top 21 genes obtained from univariate Cox analysis that were associated with prognosis. (aHR > 1 indicated poor prognosis). **(B)** By setting the lambda.min = 0.017 for LASSO regression curve, we obtained 10 prognostic-related genes(up). Each line depicted the coefficient assigned to a distinct variable, selected for its significant prognostic value. (bottom). **(C)** The forest plot displayed the top 10 genes obtained from multivariate Cox analysis that were associated with prognosis. (aHR > 1 indicated poor prognosis). **(D)** The bar plot showed gene coefficients about those 10 prognostic-related genes. **(E)** A curve graph compared the risk scores between patients in the low and high STRS groups, while a scatter plot visualized survival outcomes, with blue dots indicating survival events and red dots signifying death events. **(F)** The heatmap contrasted the expression levels of ten risk genes between the high and low STRS groups, providing insights into their differential activation patterns. **(G)** The ROC curve analysis, along with its corresponding AUC value, offered a quantitative assessment of the predictive performance of the model in estimating patient survival cycles. **(H)** A Kaplan-Meier survival analysis was conducted to compare the survival outcomes between patients in the high STRS group and those in the low STRS group. **(I)** A Kaplan-Meier survival analysis distinguished survival trends between patients stratified into high *KDELR2* group and low *KDELR2* group.

To investigate the prognostic factors in patients, we employed univariate Cox regression analysis to discover 21 genes that exhibited an association with prognosis (**Figure 8A**). We could observe *HES1* HR<1, while HR values of other genes were>1. Therefore, *HES1* was a protective factor that favors patient prognosis, while others were risk factors and unfavorable to patient prognosis. To mitigate the issue of multicollinearity among genes, we employed LASSO regression analysis to identify 10 genes that were associated with prognosis (**Figure 8B**). Multivariate Cox regression analysis was then performed on the above genes and used to calculate the genes risk coefficient for these genes (**Figures 8C, D**). The curve graph and scatter plot revealed variability in risk scores and survival outcomes between low STRS (*S100A8*+ tumor risk score) group and high STRS group, while high STRS group was associated with worse outcome (**Figure 8E**). The heatmap showcased the distinctive expression patterns of the prognostic genes in both cohorts, setting them apart from the rest of the gene pool (**Figure 8F**), *HES1* was more significantly expressed in low STRS group and *HES1* favors the prognosis of the patient, consistent with previous conclusions. The ROC curves and AUC values (**Figure 8G**) for 1-year, 3-years, and 5-years periods exceeded the threshold of 0.6, indicating that the prediction model was specific and valuable. The Kaplan-Meier survival curve further confirmed the low survival profile of high STRS group (**Figure 8H**). Combined with **Figure 8D**, the *KDELR2* risk score was the highest. Hence, we conducted an analysis using the Kaplan-Meier survival curve (**Figure 8I**) and observed a notable decline in survival rates among patients exhibiting elevated *KDELR2* gene expression.

### 3.9 Prognostic model enrichment analysis

**Figure 9:**
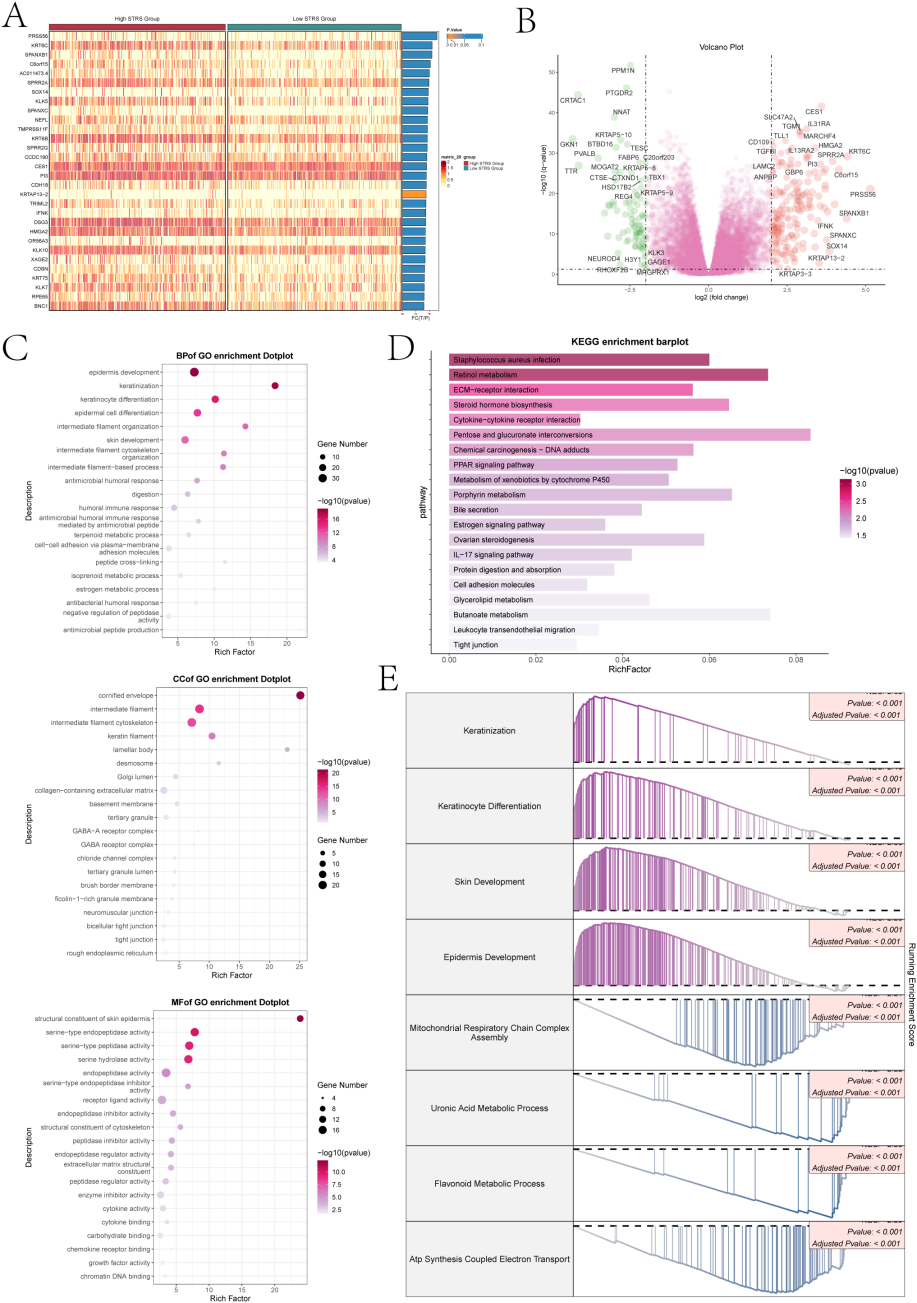
Differential gene expression and enrichment analysis. **(A)** The heatmap illustrated distinct patterns of gene expression between the high and low STRS groups. **(B)** The volcano plot visually displayed the variation in expression levels among genes that exhibited differential expression. **(C)** The dot plots sequentially displayed Biological Process (BP), Cellular Component (CC), and Molecular Function (MF) categories from the GO enrichment analysis. **(D)** KEGG enrichment bar plot showed top 20 enrichment pathways. **(E)** Eight GSEA pathways that were positively and negatively enriched.

To provide clarity on the distinction between the two scoring cohorts, an analysis was conducted on the genes that exhibited differential expression (**Figure 9A**). The volcano plot showed the up-regulation and downregulation trends of DEGs (**Figure 9B**). Subsequently, to understand the biological processes for the above genes, we performed various enrichment analyses. The first was GO enrichment analysis, which reveals the main biological process, cell composition and molecular function (**Figure 9C**). In GO-BP, genes were mainly enriched in biological processes like epidermis development, keratinization, keratinocyte differentiation, intermediate filament organization, skin development. In GO-CC, its predominant enrichment were observed in the cornified envelope, intermediate filament, and cytoskeletal components of intermediate filaments as well as keratin filament. In GO-MF, mainly enriched in structural constituent of skin epidermis, serine-type endopeptidase activity, serine hydrolase activity, endopeptidase activity and receptor ligand activity. In addition, we performed KEGG pathway enrichment analysis of differential genes (**Figure 9D**), and showed that the related pathways were staphylococcus aureus infection, retinol metabolism, ECM-receptor interaction, steroid hormone biosynthesis and cytokine-cytokine receptor interaction. Finally, we performed a GSEA of the enriched pathways (**Figure 9E**), the results showed that keratinization, keratinocyte differentiation, skin development and the epidermis development related pathways showed a positive enrichment trend, mitochondrial respiratory chain complex assembly, uronic acid metabolic process, the flavonoid metabolic process and ATP synthesis coupled electron transport related pathways showed a negative enrichment trend.

### 3.10 Disparities in immune infiltration observed between high and low STRS groups

**Figure 10:**
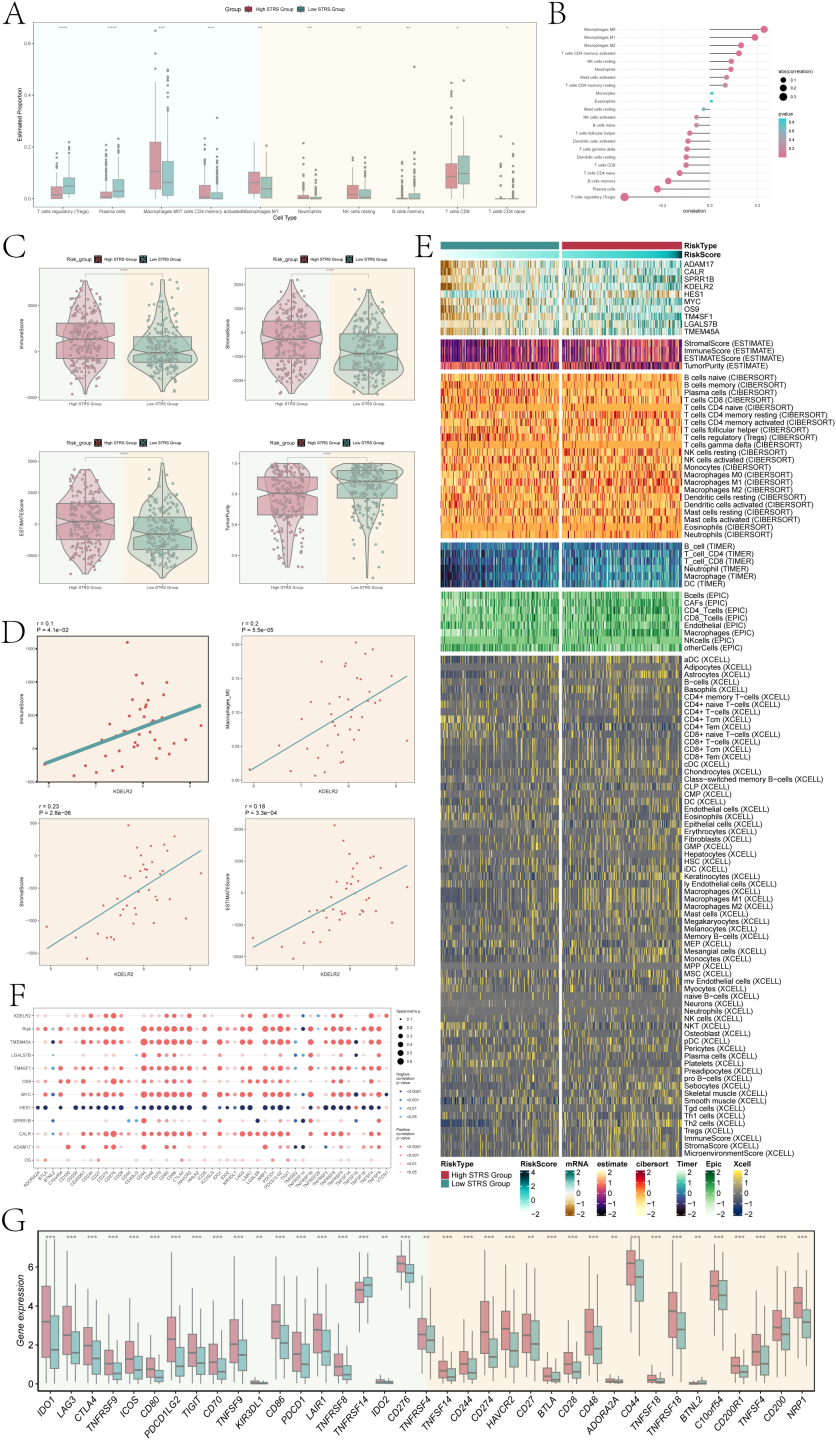
Infiltration of immune cells in high STRS group and low STRS group. **(A)** The box plot showed estimated proportion of immune cells that were statistically different between high STRS group and low STRS group. **(B)** Lollipop chart showed the correlation between different immune pathways and risk scores, with bubble size representing the abs(correlation) and color indicating p-value. **(C)** Immune-score, stromal-score, ESTIMATE-score, tumor-purity between high STRS group and low STRS group. **(D)** The scatter diagram showed the correlation among *KDELR2* and immune-score, macrophages-M0, stromal-score, ESTIMATE-score. **(E)** The heatmap showed risk scores for different immune cells in the high STRS group and the low STRS group. **(F)** The bubble plot showed the degree of association between risk genes and immune checkpoints. **(G)** The box plot showed immune checkpoints with statistical differences in the high STRS group and the low STRS group. *P < 0.05, **P < 0.01, ***P < 0.001 and ****P < 0.0001.

We performed immune infiltration analysis on high STRS group and low STRS group. The box plot revealed higher expression levels of macrophages M0 and M1, and neutrophils in the high STRS group (**Figure 10A**). Those cell types belong to myeloid cells. It was noteworthy that macrophages exhibited a higher abundance of immune infiltration and exerted a more pronounced effect on the high STRS group. Subsequently, we further investigated the correlation between risk score and immune infiltration (**Figure 10B**). In the correlation lollipop chart, macrophages also showed a close positive correlation with risk score. In addition, the immune-score, stromal-score, and ESTIMATE-score of the high STRS group were significantly higher than those of the low STRS group, while the tumor-purity was significantly lower than that of the low STRS group (**Figure 10C**). Scatter plots demonstrated positive associations between *KDELR2* expression levels with immune-score, stromal-score, and ESTIMATE-score as well as macrophages M0 (**Figure 10D**). Elevated *KDELR2* expression was linked to increased infiltration of macrophages and a more prominent immunosuppressive microenvironment. Therefore, target mining of macrophages might be a potential strategy for bladder cancer immunotherapy. The heatmap provided visual comparisons that supported our previous findings (**Figure 10E**).

Furthermore, the bubble plot (**Figure 10F**) showed the correlation between immune checkpoints and prognostic genes, OS, Risk. Among them, CD274, CD276, CD80, CD86, HAVCR2, LAIR1, NRP1, PDCD1LG2, TNFRSF8, TNFRSF9, TNFRSF4, and VTCN1 were significantly positively correlated with *KDELR2*. This suggested that patients with high *KDELR2* expression were potential candidates for these immune checkpoint inhibitors (ICIs). In order to optimize ICIs utilization and improve therapeutic efficacy further analysis was conducted on gene expression levels of different immune checkpoints between high STRS group and low STRS group using a box plot (**Figure 10G**). The results indicated significantly higher expression levels for most genes in the high STRS group compared to those in the low STRS group. These findings suggested that application of these ICIs to patients classified as high STRS group might result in improved treatment outcomes.

### 3.11 *KDELR2* inhibits the proliferation and invasion of bladder cancer cells

**Figure 11:**
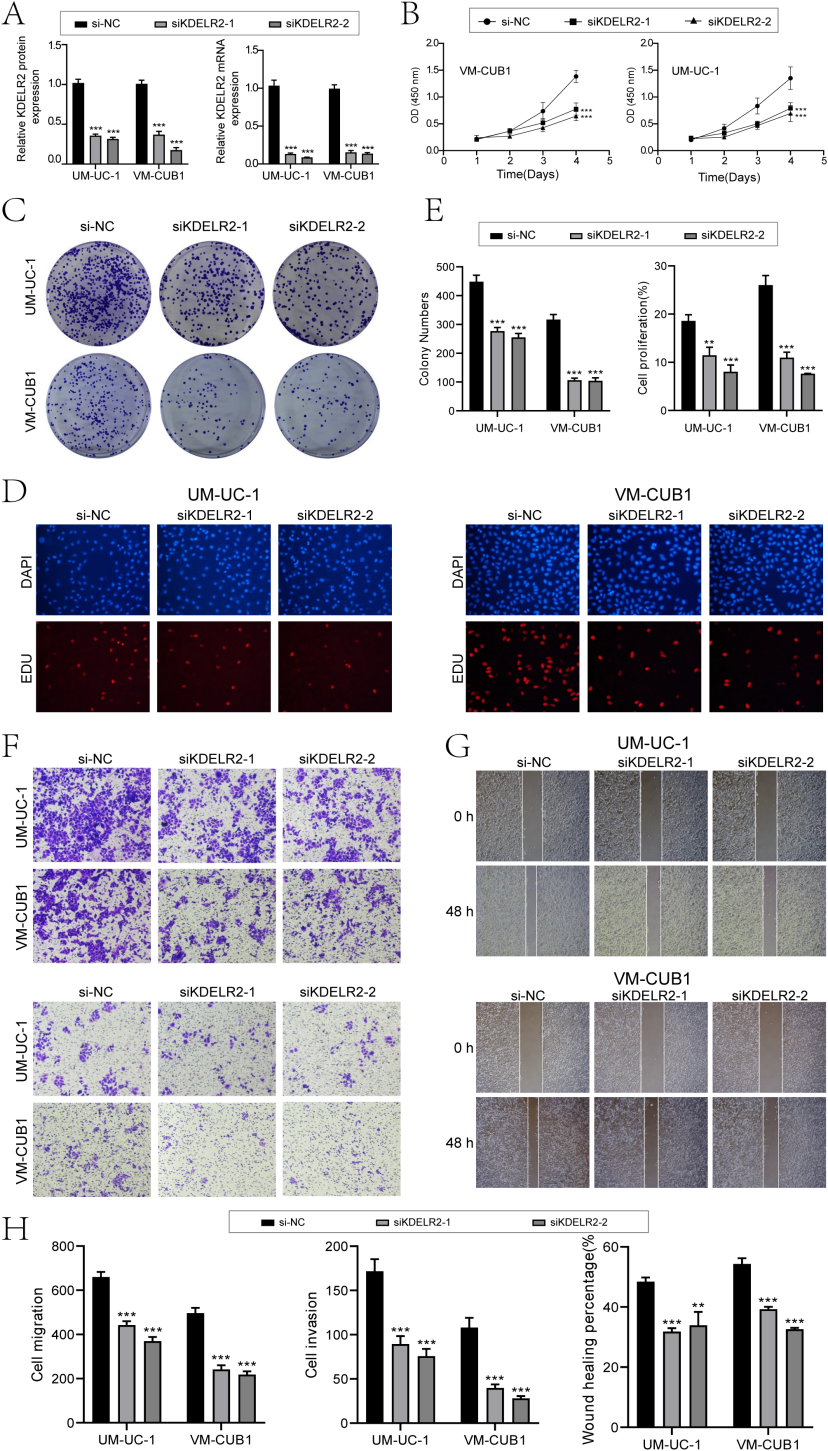
*In vitro* experiments confirmed the effects of *KDELR2* knockdown. **(A)** The bar charts depicted the altered patterns of gene-encoded protein (left) and gene RNA (right) expression in UM-UC-1 and VM-CUB1 cell lines, comparing three groups: si-NC, si*KDELR2*-1, and si*KDELR2*-2. Following targeted *KDELR2* knockdown, notable reductions in both mRNA and protein abundance levels were evident. **(B)** The line plot showed the longitudinal growth of three distinct groups across two cell lines. **(C)** Colony-formation assay revealed a significant reduction in cell viability subsequent to *KDELR2* knockdown, in contrast to the unaltered control group. **(D)** The EDU staining assay confirmed that *KDELR2* knockdown exerted an inhibitory effect on cell proliferation. **(E)** The bar plots showed the colony numbers and cell proliferation of three groups in two cell lines. **(F)** The transwell assay assessed the migratory and invasive potential of three distinct groups across two cellular lines, offering quantitative insights into their motility and aggressiveness. **(G)** Post-treatment migration capacity of TCs was quantitatively assessed using wound healing assays. **(H)** *KDELR2* knockdown led to a statistically significant decrease in cell migration, invasion, and wound healing capacities, as evident from bar graph analysed. **P < 0.01, ***P < 0.001.

In an effort to delve deeper into the impact of *KDELR2* on the patient outcome in cases of bladder cancer, we conducted *in vitro* experiments. Firstly, we divided tumor cell lines of different sources into three groups: si-NC, si-*KDELR2*-1 and si-*KDELR2*-2. The findings indicated notable variances in the expression levels of protein and mRNA for UM-UC-1 and VM-CUB1. Specifically, the si-*KDELR2* group exhibited lower expression levels compared to the si-NC group (**Figure 11A**). In addition, we investigated the effect of *KDELR2* on tumor cell activity. First, tumor cell activity was tested by CCK-8, and fluorescence staining showed that the tumor cell activity in si-NC was higher than that in si-*KDELR2*-1 and si-*KDELR2*-2. Subsequently, we used line plots to quantify indicators. The OD values of si-*KDELR2*-1 and si-*KDELR2*-2 were found to be significantly reduced compared to the si-NC group, aligning with the outcomes obtained from CCK-8 staining (**Figure 11B**). Next, we studied the proliferation ability of TCs. The colony formation assay (**Figure 11C**) similarly corroborated the aforementioned statements. Through EDU experiments, the microbiota density was lower after *KDELR2* was knocked out. Subsequent data analysis showed that si-*KDELR2* inhibited cell proliferation and cloning (**Figures 11D, E**). In order to investigate the invasion and migration ability of the cells, we conducted wound healing assay and transwell assay (**Figures 11F, G**). After 48 hours of assay, si-*KDELR2* TCs’ distance was greater than that of si-NC, while the density was lower than that of si-NC, which proved that si-*KDELR2* TCs had low invasion and migration ability. The bar plots were consistent with the experimental conclusion that UM-UC-1 and VM-CUB1 cells inhibited the invasion, migration and wound healing of TCs after the knockout of si-*KDELR2* (**Figure 11H**). In summary, *KDELR2* had obvious tumor promoting effect, which played a key role in the development of TCs. In conclusion, our research provided a more comprehensive understanding of the *KDELR2* gene. Through a series of studies, we discovered that *KDELR2* promoted tumor cell development. It could be inferred that *KDELR2* played a key role in driving tumor cell proliferation, migration, and other related processes. Accordingly, we could achieve the purpose of improving prognostic survival by inhibiting *KDELR2*, making it possible focus for therapeutic intervention in cases of bladder cancer.

## 4 Discussion

In today’s society, bladder cancer is still a global problem, posing a threat to human health, and its incidence has significant population differences, the incidence of men is generally higher than that of women, but most female patients with bladder cancer are more serious and have a poor prognosis (55). In most cases, the prognosis of bladder cancer is determined by the distinct attributes and differences observed among individual patients (56). Hence, it is imperative to conduct a thorough investigation into the diversity of bladder cancer in order to improve patient prognosis and identify possible targets for therapeutic interventions in this disease. Using scRNA-seq, the cellular and molecular features of bladder cancer tissues and identified five known cell types. For different cell subpopulations, we performed temporal phase, sample source as well as pathway enrichment analysis. We found that unlike other cells, EPCs were mainly derived from bladder cancer tissue cells.

Enrichment analysis showed that ATP synthesisation-related pathways were significantly expressed in EPCs, and Mitochondrial electron transport and NADH to ubiquinone were positively enriched in EPCs. ATP played a very important role in cells and was one of the important links of energy information, which could control the life activity of tumors (57)and make the adverse process, namely tumor proliferation, possible, thus promoting the development of TCs (58). Mitochondria could provide an energy source for cells, and in cells they could influence signature functions, including avoiding cell death, bioenergy dysregulation, genome mutation, and promoting tumor inflammation and metastasis (59). Cancer cells would derive a kind of tumor stem cells with the ability of self-renewal, metastasis and spread and treatment resistance during their self-evolution, which played a key role in tumor occurrence and development (60). Based on this, we studied TCs of bladder cancer. First, tumor tissue was divided into six subpopulations based on marker genes. Among them, we discovered that TCs exhibiting elevated levels of *S100A8* were exclusively derived from tumor tissue specimens. Bubble chart showed the C1 subpopulation high expressed *DMKN MT1X, S100A7, S100A9, S100A8* genes, etc. Among them, *S100A9* and *S100A8* had strong pro-inflammatory functions. According to their high expression in cancer, they showed abundant expression in TCs and infiltrate immune cells. The involvement of these factors was crucial in the progression of cancer. Furthermore, it was observed that C1 subpopulation had higher expression levels of CNV score and Cell-stemness. Therefore, we could conclude that C1 subpopulation cells have higher malignant degree and higher differentiation potential. Overall, C1 *S100A8*+ TCs had complex relationships with further development of bladder cancer.

To clarify the relationship between C1 *S100A8*+ TCs subpopulation and tumor, we performed various enrichment analyses on cell subpopulations. C1 *S100A8*+ TCs were mainly involved in the cytoplasmic translation, protein folding, chaperone-mediated protein folding and signaling pathways like ribonucleoprotein complex biogenesis. Studies had shown that there was a special class of ribosomes in cancer cells, which could change cell progression and metabolic reprogramming, accelerate carcinogenic translation, and change cell function (61). In other words, bladder cancer cells might develop more rapidly under the support of ribosomes, and correspondingly, their malignancy might be higher. In addition, protein folding tends to have a relationship with the endoplasmic reticulum. When the body’s ability to tolerate ERS was improved, the survival ability of cancer cells will become stronger. Similarly, the ability of immunosuppression, angiogenesis and drug resistance would be improved accordingly (62). To further enhanced our understanding of ribosome function and protein folding, we focused on their critical roles in cancer drug resistance and tumor evolution. Ribosomes played a vital role in maintaining cellular proteostasis, and alterations in ribosomal biogenesis enhanced protein synthesis capacity, allowing cancer cells to adapt to therapeutic pressure (63). Additionally, the involvement of chaperone proteins and the accumulation of misfolded proteins triggered the unfolded protein response (64), activating autophagy or heat shock protein-mediated survival pathways, thereby promoting resistance to chemotherapy and targeted therapies. Defects in protein folding also led to the accumulation of mutations, exacerbating tumor heterogeneity and evolution (65), and affected tumor antigen presentation, potentially resulting in immune evasion. Moreover, ribosomal alterations impacted translational fidelity, increasing genetic and phenotypic diversity, further driving tumor adaptation to the microenvironment and therapeutic interventions (66). In summary, ribosome and protein folding and other related biological processes played an important role in tumor development, which can accelerate the proliferation and metastasis of TCs. In simpler terms, the progression of bladder cancer could be hindered by targeting specific factors like ribosomes and the process of protein folding.

To further explore the interaction between C1 *S100A8*+ TCs and other cells, CellChat found that C1 *S100A8*+ TCs acted on myeloid cells through the MIF-(CD74+CXCR4) and MIF-(CD74+CD44) signaling pathways. MIF was a representative pro-inflammatory factor that played a role in regulating immune responses. Studies had shown that MIF expression is significantly increased in a variety of tumors (67). High expression of MIF had been observed to promote tumor progression and metastasis, stimulate angiogenesis, and create an immunosuppressive microenvironment conducive to tumor development. C1 *S100A8*+ TCs acted on myeloid cells CD74-CXCR4 and CD74-CD44 receptors through MIF ligands, which might promote the transformation of normal myeloid cells into cancer-related myeloid cells and inhibit the body’s normal anti-tumor immune effect. Therefore, targeting MIF in TCs could be used as a potential strategy to treat tumors (67).

To further elucidate the mechanism of carcinogenesis in the subpopulation of C1 *S100A8*+ TCs, we analyzed the TFs and selected the top five TFs according to the expression active, namely HES1, AHR, TBL1XR1, IRF4 and SPI1.We could find that HES1 and AHR were significantly expressed in C1 subpopulation. HES1 had a complex relationship with various pathways, which had the potential to trigger cellular metamorphosis and enhance its invasive capabilities, while also playing a significant role in the differentiation, proliferation, and immune suppression of cancer cells (68). Furthermore, research had indicated a strong correlation between HES1 and the stem-like properties of TCs, as well as their ability to spread and develop resistance against drugs (69). AHR activated a specific gene, *CYP1A1*, which caused many oncogenic genes to combine with DNA to form cancer-promoting combinations, thus promoting the development of cancer (70). The over-expression or abnormal activation of AHR or its endogenous agonists in the TME, and these regulatory abnormalities promoted the immune escape of tumors. In summary, the above studies provided innovative prospects for future immunotherapeutic interventions in bladder cancer.

In addition, to enhance the chances of survival and extend the lifespan of individuals and improve the quality of life of patients, our research group constructed a bladder cancer prognostic risk model based on the top 10 marker genes of C1 *S100A8*+ TCs, and evaluated the prognosis of patients characterized by high expression of *S100A8* in the TCGA cohort. We observed that a higher high STRS group risk score, often symbolizing higher mortality, had a worse prognosis than low STRS group. According to research findings, *KDELR2* had been found to contribute to the advancement of bladder cancer and was often associated with unfavorable outcomes in individuals who had been diagnosed with cancer (71). In vitro experiments had also proved that when *KDELR2* was highly expressed, the proliferation, migration and invasion ability of TCs were significantly improved, while when *KDELR2* was knocked down, it had the opposite effect. This was consistent with previous studies (5, 72). Therefore, the potential of targeting *KDELR2* should be acknowledged as a viable strategy in the management of patients diagnosed with bladder cancer.

Additionally, the results of immune cell infiltration analysis showed that the high STRS group had higher macrophage infiltration levels, and the immune-score and stromal-score were significantly higher than those of the low STRS group. Macrophages have two phenotypes: “anti-tumor M1” and “pro-tumor M2”, which respectively performed two opposite biological effects (73, 74). Therefore, reshaping the macrophage phenotype and promoting the transformation of the M0 phenotype to the M1 direction was crucial to resist tumor immunosuppression. The higher macrophage infiltration level in the high STRS group was obviously beneficial for macrophage-targeted therapy in bladder cancer patients to help promote the normal anti-tumor effect of macrophages. In addition, with the increase of *KDELR2* expression level, the macrophage M0 immune infiltration status became more obvious, and both immune and stromal scores increased. *KDELR2* could enhance tumor immune infiltration, inhibit anti-tumor immune response, and lead to low response to immunotherapy in patients (71). *KDELR2* was instrumental in promoting macrophage infiltration into the TME of bladder cancer, where it influenced the polarization of macrophages towards the M2 phenotype (75). This M2 polarization was associated with immunosuppression, increased angiogenesis, and enhanced tumor survival, creating a favorable environment for tumor progression (76). By modulating cytokine and chemokine production, *KDELR2* helped recruit macrophages that secreted factors supporting tumor growth. Additionally, the presence of M2 macrophages facilitated immune evasion, allowing cancer cells to escape detection by the immune system. Thus, targeting *KDELR2* presented a promising therapeutic strategy to disrupt these pro-tumorigenic processes and improve treatment outcomes in bladder cancer.

To further elucidate the modulation of macrophage phenotypes for the purpose of anti-tumor therapy, particularly focusing on the conversion of M2 macrophages to the pro-inflammatory M1 phenotype, previous studies showed promise in reprogramming macrophages toward an anti-tumor state using Toll-Like Receptor agonists, such as MPLA (77), and CSF1R inhibitors, such as pexidartinib (78, 79). Additionally, researchers explored targeting the STAT3 (80) and PI3K/AKT signaling pathways (81) to suppress M2 polarization while promoting M1 activity. Cytokine-based therapies, including IFN-γ and IL-12 (82), were also employed to enhance the M1 phenotype. Our findings on the role of *KDELR2* in macrophage polarization potentially complemented these therapeutic strategies, as *KDELR2* might have represented a novel target for further enhancing macrophage reprogramming. Future studies could have explored combining *KDELR2* targeting with these existing therapies to improve the efficacy of macrophage-based immunotherapies in bladder cancer.

In our study, although we made every effort to conduct a thorough analysis and carefully selected the study cohort, we acknowledged several limitations. First, the small sample size might have led to an erroneous association between the investigated samples and the target genes, potentially affecting the accuracy of our assessment. Second, bladder cancer was composed of various subpopulations. Due to the relative rarity of bladder squamous cell carcinoma and adenocarcinoma, we focused primarily on urothelial carcinoma in our study. This focus might have limited the generalizability of our findings, as different subpopulations of bladder cancer might exhibit distinct biological behaviors and treatment responses. Lastly, our experimental investigation into the interactions between tumor cells and macrophages was not sufficiently in-depth. The complexity of the TME, especially the interactions between tumor cells and immune cells, was a current research hotspot. In bladder cancer, the cellular components of the TME, such as tumor-associated macrophages and cancer-associated fibroblasts, co-evolve with tumor cells, contributing to tumor heterogeneity and promoting tumor progression and drug resistance. Therefore, future studies need to focus more on these interactions and how they influence tumor progression and treatment response.

In future research, we plan to start with *KDELR2* and further investigate macrophage-targeted therapeutic strategies for bladder cancer. By exploring *KDELR2*’s mechanisms in bladder cancer, we aim to develop novel therapeutic strategies to improve patient prognosis. Additionally, we will explore combining different TME-targeting strategies into a rational approach that offers better efficacy with fewer side effects, providing more treatment options for bladder cancer patients.

## Data Availability

The original contributions presented in the study are included in the article/**Supplementary Material**. Further inquiries can be directed to the corresponding authors.

## Supplementary material

Supplementary Figure 1 The heatmaps showed the scores of CNV for ECs, EPCs, and six tumor cell clusters. **(A)** The heatmaps showed the scores of CNV for ECs and EPCs. **(B)** The heatmaps showed the scores of CNV for ECs and six tumor cell clusters.

## References

[1] Dobruch J Daneshmand S Fisch M Lotan Y Noon AP Resnick MJ. Gender and Bladder Cancer: A Collaborative Review of Etiology, Biology, and Outcomes. Eur Urol. (2016) 69:300–10. doi: 10.1016/j.eururo.2015.08.037 26346676

[2] Lenis AT Lec PM Chamie K Mshs MD. Bladder Cancer: A Review. Jama. (2020) 324:1980–91. doi: 10.1001/jama.2020.17598 33201207

[3] Whitmore WJ. Bladder cancer: an overview. CA Cancer J Clin. (1988) 38:213–23. doi: 10.3322/canjclin.38.4.213 3135080

[4] Chang SS Bochner BH Chou R Dreicer R Kamat AM Lerner SP. Treatment of Non-Metastatic Muscle-Invasive Bladder Cancer: AUA/ASCO/ASTRO/SUO Guideline. J Urol. (2017) 198:552–59. doi: 10.1016/j.juro.2017.04.086 PMC5626446 28456635

[5] Wei H Ma W Lu X Liu H Lin K Wang Y. KDELR2 promotes breast cancer proliferation via HDAC3-mediated cell cycle progression. Cancer Commun (Lond). (2021) 41:904–20. doi: 10.1002/cac2.12180 PMC8441056 34146461

[6] Sun L Shao W Lin Z Lin J Zhao F Yu J. Single-cell RNA sequencing explored potential therapeutic targets by revealing the tumor microenvironment of neuroblastoma and its expression in cell death. Discovery Oncol. (2024) 15:409. doi: 10.1007/s12672-024-01286-5 PMC11377405 39235657

[7] Zhao F Hong J Zhou G Huang T Lin Z Zhang Y. Elucidating the role of tumor-associated ALOX5+ mast cells with transformative function in cervical cancer progression via single-cell RNA sequencing. Front Immunol. (2024) 15:1434450. doi: 10.3389/fimmu.2024.1434450 39224598 PMC11366577

[8] Shao W Lin Z Xiahou Z Zhao F Xu J Liu X. Single-cell RNA sequencing reveals that MYBL2 in malignant epithelial cells is involved in the development and progression of ovarian cancer. Front Immunol. (2024) 15:1438198. doi: 10.3389/fimmu.2024.1438198 39136009 PMC11317301

[9] Zhang Y Zhao Z Huang W Kim BS Lin L Li X. Pan-Cancer Single-Cell Analysis Revealing the Heterogeneity of Cancer-Associated Fibroblasts in Skin Tumors. Curr Gene Ther. (2024). doi: 10.2174/0115665232331353240911080642 39323331

[10] Nie W Zhao Z Liu Y Wang Y Zhang J Hu Y. Integrative Single-Cell Analysis of Cardiomyopathy Identifies Differences in Cell Stemness and Transcriptional Regulatory Networks among Fibroblast Subpopulations. Cardiol Res Pract. (2024) 2024:3131633. doi: 10.1155/2024/3131633 38799173 PMC11127766

[11] Ding Y Zhao Z Cai H Zhou Y Chen H Bai Y. Single-cell sequencing analysis related to sphingolipid metabolism guides immunotherapy and prognosis of skin cutaneous melanoma. Front Immunol. (2023) 14:1304466. doi: 10.3389/fimmu.2023.1304466 38077400 PMC10701528

[12] Chen N Fan B He Z Yu X Wang J. Identification of HBEGF+ fibroblasts in the remission of rheumatoid arthritis by integrating single-cell RNA sequencing datasets and bulk RNA sequencing datasets. Arthritis Res Ther. (2022) 24:215. doi: 10.1186/s13075-022-02902-x 36068607 PMC9446562

[13] Korsunsky I Millard N Fan J Slowikowski K Zhang F Wei K. Fast, sensitive and accurate integration of single-cell data with Harmony. Nat Methods. (2019) 16:1289–96. doi: 10.1038/s41592-019-0619-0 PMC6884693 31740819

[14] Ge Q Zhao Z Li X Yang F Zhang M Hao Z. Deciphering the suppressive immune microenvironment of prostate cancer based on CD4+ regulatory T cells: Implications for prognosis and therapy prediction. Clin Transl Med. (2024) 14:e1552. doi: 10.1002/ctm2.1552 38239097 PMC10797244

[15] Li X Lin Z Zhao F Huang T Fan W Cen L. Unveiling the cellular landscape: insights from single-cell RNA sequencing in multiple myeloma. Front Immunol. (2024) 15:1458638. doi: 10.3389/fimmu.2024.1458638 39281682 PMC11392786

[16] Zhou W Lin Z Tan W. Deciphering the molecular landscape: integrating single-cell transcriptomics to unravel myofibroblast dynamics and therapeutic targets in clear cell renal cell carcinomas. Front Immunol. (2024) 15:1374931. doi: 10.3389/fimmu.2024.1374931 38562930 PMC10982338

[17] Liu P Xing N Xiahou Z Yan J Lin Z Zhang J. Unraveling the intricacies of glioblastoma progression and recurrence: insights into the role of NFYB and oxidative phosphorylation at the single-cell level. Front Immunol. (2024) 15:1368685. doi: 10.3389/fimmu.2024.1368685 38510250 PMC10950940

[18] Lin Z Sui X Jiao W Chen C Zhang X Zhao J. Mechanism investigation and experiment validation of capsaicin on uterine corpus endometrial carcinoma. Front Pharmacol. (2022) 13:953874. doi: 10.3389/fphar.2022.953874 36210802 PMC9532580

[19] Lin Z Li X Shi H Cao R Zhu L Dang C. Decoding the tumor microenvironment and molecular mechanism: unraveling cervical cancer subpopulations and prognostic signatures through scRNA-Seq and bulk RNA-seq analyses. Front Immunol. (2024) 15:1351287. doi: 10.3389/fimmu.2024.1351287 38482016 PMC10933018

[20] Xing J Cai H Lin Z Zhao L Xu H Song Y. Examining the function of macrophage oxidative stress response and immune system in glioblastoma multiforme through analysis of single-cell transcriptomics. Front Immunol. (2023) 14:1288137. doi: 10.3389/fimmu.2023.1288137 38274828 PMC10808540

[21] Jin W Zhang Y Zhao Z Gao M. Developing targeted therapies for neuroblastoma by dissecting the effects of metabolic reprogramming on tumor microenvironments and progression. Theranostics. (2024) 14:3439–69. doi: 10.7150/thno.93962 PMC11209723 38948053

[22] Zhao Z Ding Y Tran LJ Chai G Lin L. Innovative breakthroughs facilitated by single-cell multi-omics: manipulating natural killer cell functionality correlates with a novel subcategory of melanoma cells. Front Immunol. (2023) 14:1196892. doi: 10.3389/fimmu.2023.1196892 37435067 PMC10332463

[23] Huang W Kim BS Zhang Y Lin L Chai G Zhao Z. Regulatory T cells subgroups in the tumor microenvironment cannot be overlooked: Their involvement in prognosis and treatment strategy in melanoma. Environ Toxicol. (2024) 39:4512–30. doi: 10.1002/tox.24247 38530049

[24] Zhao ZJ Zheng RZ Wang XJ Li TQ Dong XH Zhao CY. Integrating Lipidomics and Transcriptomics Reveals the Crosstalk Between Oxidative Stress and Neuroinflammation in Central Nervous System Demyelination. Front Aging Neurosci. (2022) 14:870957. doi: 10.3389/fnagi.2022.870957 35547618 PMC9083465

[25] Tang C Deng L Luo Q He G. Identification of oxidative stress-related genes and potential mechanisms in atherosclerosis. Front Genet. (2022) 13:998954. doi: 10.3389/fgene.2022.998954 36685865 PMC9845256

[26] Zhao Z Luo Q Liu Y Jiang K Zhou L Dai R. Multi-level integrative analysis of the roles of lncRNAs and differential mRNAs in the progression of chronic pancreatitis to pancreatic ductal adenocarcinoma. BMC Genomics. (2023) 24:101. doi: 10.1186/s12864-023-09209-4 36879212 PMC9990329

[27] Lin Z Fan W Yu X Liu J Liu P. Research into the mechanism of intervention of SanQi in endometriosis based on network pharmacology and molecular docking technology. Med (Baltimore). (2022) 101:e30021. doi: 10.1097/MD.0000000000030021 PMC9478308 36123943

[28] Lin Z Sui X Jiao W Wang Y Zhao J. Exploring the mechanism and experimental verification of puerarin in the treatment of endometrial carcinoma based on network pharmacology and bioinformatics analysis. BMC Complement Med Ther. (2022) 22:150. doi: 10.1186/s12906-022-03623-z 35672846 PMC9175360

[29] Zou J Lin Z Jiao W Chen J Lin L Zhang F. A multi-omics-based investigation of the prognostic and immunological impact of necroptosis-related mRNA in patients with cervical squamous carcinoma and adenocarcinoma. Sci Rep. (2022) 12:16773. doi: 10.1038/s41598-022-20566-0 36202899 PMC9537508

[30] Lin Z Fan W Sui X Wang J Zhao J. Necroptosis-Related LncRNA Signatures for Prognostic Prediction in Uterine Corpora Endometrial Cancer. Reprod Sci. (2023) 30:576–89. doi: 10.1007/s43032-022-01023-9 PMC9988759 35854199

[31] Lin Z Zou J Sui X Yao S Lin L Wang J. Necroptosis-related lncRNA signature predicts prognosis and immune response for cervical squamous cell carcinoma and endocervical adenocarcinomas. Sci Rep. (2022) 12:16285. doi: 10.1038/s41598-022-20858-5 36175606 PMC9523019

[32] Zhao J Zou J Jiao W Lin L Wang J Lin Z. Construction of N-7 methylguanine-related mRNA prognostic model in uterine corpus endometrial carcinoma based on multi-omics data and immune-related analysis. Sci Rep. (2022) 12:18813. doi: 10.1038/s41598-022-22879-6 36335189 PMC9637130

[33] Zhao Z Li T Dong X Wang X Zhang Z Zhao C. Untargeted Metabolomic Profiling of Cuprizone-Induced Demyelination in Mouse Corpus Callosum by UPLC-Orbitrap/MS Reveals Potential Metabolic Biomarkers of CNS Demyelination Disorders. Oxid Med Cell Longev. (2021) 2021:7093844. doi: 10.1155/2021/7093844 34567412 PMC8457991

[34] Wang Y Zhao ZJ Kang XR Bian T Shen ZM Jiang Y. lncRNA DLEU2 acts as a miR-181a sponge to regulate SEPP1 and inhibit skeletal muscle differentiation and regeneration. Aging (Albany NY). (2020) 12:24033–56. doi: 10.18632/aging.104095 PMC7762514 33221762

[35] Zhao ZJ Chen D Zhou LY Sun ZL Wang BC Feng DF. Prognostic Value of Different Computed Tomography Scoring Systems in Patients With Severe Traumatic Brain Injury Undergoing Decompressive Craniectomy. J Comput Assist Tomogr. (2022) 46:800–07. doi: 10.1097/RCT.0000000000001343 35650015

[36] Zhao ZJ Wei DP Zheng RZ Peng T Xiao X Li FS. The Gene Coexpression Analysis Identifies Functional Modules Dynamically Changed After Traumatic Brain Injury. Comput Math Methods Med. (2021) 2021:5511598. doi: 10.1155/2021/5511598 33953790 PMC8068551

[37] Zheng R Zhuang Z Zhao C Zhao Z Yang X Zhou Y. Chinese Admission Warning Strategy for Predicting the Hospital Discharge Outcome in Patients with Traumatic Brain Injury. J Clin Med. (2022) 11974,1-13. doi: 10.3390/jcm11040974 PMC8880692 35207247

[38] Yuan J Zhang J Luo Q Peng L. Effects of nonalcoholic fatty liver disease on sarcopenia: evidence from genetic methods. Sci Rep. (2024) 14:2709. doi: 10.1038/s41598-024-53112-1 38302636 PMC10834579

[39] Tang L Xu H Wu T Wu W Lu Y Gu J. Advances in tumor microenvironment and underlying molecular mechanisms of bladder cancer: a systematic review. Discovery Oncol. (2024) 15:111. doi: 10.1007/s12672-024-00902-8 PMC11009183 38602556

[40] Yang F Fang E Mei H Chen Y Li H Li D. Cis-Acting circ-CTNNB1 Promotes beta-Catenin Signaling and Cancer Progression via DDX3-Mediated Transactivation of YY1. Cancer Res. (2019) 79:557–71. doi: 10.1158/0008-5472.CAN-18-1559 30563889

[41] Paredes F Williams HC San MA. Metabolic adaptation in hypoxia and cancer. Cancer Lett. (2021) 502:133–42. doi: 10.1016/j.canlet.2020.12.020 PMC8158653 33444690

[42] Wu Q You L Nepovimova E Heger Z Wu W Kuca K. Hypoxia-inducible factors: master regulators of hypoxic tumor immune escape. J Hematol Oncol. (2022) 15:77. doi: 10.1186/s13045-022-01292-6 35659268 PMC9166526

[43] Zhao Y Yang W Zheng K Chen J Jin X. The role of BMI1 in endometrial cancer and other cancers. Gene. (2023) 856:147129. doi: 10.1016/j.gene.2022.147129 36563713

[44] Xie J Zhang W Liang X Shuai C Zhou Y Pan H. RPL32 Promotes Lung Cancer Progression by Facilitating p53 Degradation. Mol Ther Nucleic Acids. (2020) 21:75–85. doi: 10.1016/j.omtn.2020.05.019 32516735 PMC7281510

[45] Hou G Lu Z Jiang J Yang X. Ribosomal protein L32 enhances hepatocellular carcinoma progression. Cancer Med. (2023) 12:10791–803. doi: 10.1002/cam4.5811 PMC10225200 37017565

[46] Bi N Sun Y Lei S Zeng Z Zhang Y Sun C. Identification of 40S ribosomal protein S8 as a novel biomarker for alcohol−associated hepatocellular carcinoma using weighted gene co−expression network analysis. Oncol Rep. (2020) 44:611–27. doi: 10.3892/or.2020.7634 PMC7336510 32627011

[47] Tan WK Maroni R Offman J Zamani SA Sasieni PD Fitzgerald RC. Targeted Screening for Barrett's Esophagus and Esophageal Cancer: Post Hoc Analysis From the Randomized BEST3 Trial. Gastroenterology. (2024) 167:798–800. doi: 10.1053/j.gastro.2024.04.030 38718951

[48] Brouwer-Visser J Huang GS. IGF2 signaling and regulation in cancer. Cytokine Growth Factor Rev. (2015) 26:371–77. doi: 10.1016/j.cytogfr.2015.01.002 25704323

[49] Zhang L Gao S Shi X Chen Y Wei S Mi Y. NUPR1 imparts oncogenic potential in bladder cancer. Cancer Med. (2023) 12:7149–63. doi: 10.1002/cam4.5518 PMC10067104 36468653

[50] Dong Y Ma WM Shi ZD Zhang ZG Zhou JH Li Y. Role of NRP1 in Bladder Cancer Pathogenesis and Progression. Front Oncol. (2021) 11:685980. doi: 10.3389/fonc.2021.685980 34249735 PMC8261128

[51] Brewer JW. Regulatory crosstalk within the mammalian unfolded protein response. Cell Mol Life Sci. (2014) 71:1067–79. doi: 10.1007/s00018-013-1490-2 PMC11113126 24135849

[52] Clarke HJ Chambers JE Liniker E Marciniak SJ. Endoplasmic reticulum stress in malignancy. Cancer Cell. (2014) 25:563–73. doi: 10.1016/j.ccr.2014.03.015 24823636

[53] Dai X Zhu M. Coupling of Ribosome Synthesis and Translational Capacity with Cell Growth. Trends Biochem Sci. (2020) 45:681–92. doi: 10.1016/j.tibs.2020.04.010 32448596

[54] Xiong S Dong L Cheng L. Neutrophils in cancer carcinogenesis and metastasis. J Hematol Oncol. (2021) 14:173. doi: 10.1186/s13045-021-01187-y 34674757 PMC8529570

[55] Dyrskjot L Hansel DE Efstathiou JA Knowles MA Galsky MD Teoh J. Bladder cancer. Nat Rev Dis Primers. (2023) 9:58. doi: 10.1038/s41572-023-00468-9 37884563 PMC11218610

[56] Meeks JJ Al-Ahmadie H Faltas BM Taylor JR Flaig TW DeGraff DJ. Genomic heterogeneity in bladder cancer: challenges and possible solutions to improve outcomes. Nat Rev Urol. (2020) 17:259–70. doi: 10.1038/s41585-020-0304-1 PMC7968350 32235944

[57] Shukla S Dalai P Agrawal-Rajput R. Metabolic crosstalk: Extracellular ATP and the tumor microenvironment in cancer progression and therapy. Cell Signal. (2024) 121:111281. doi: 10.1016/j.cellsig.2024.111281 38945420

[58] Vander HM DeBerardinis RJ. Understanding the Intersections between Metabolism and Cancer Biology. Cell. (2017) 168:657–69. doi: 10.1016/j.cell.2016.12.039 PMC5329766 28187287

[59] Giampazolias E Tait SW. Mitochondria and the hallmarks of cancer. FEBS J. (2016) 283:803–14. doi: 10.1111/febs.13603 26607558

[60] Najafi M Mortezaee K Ahadi R. Cancer stem cell (a)symmetry & plasticity: Tumorigenesis and therapy relevance. Life Sci. (2019) 231:116520. doi: 10.1016/j.lfs.2019.05.076 31158379

[61] Elhamamsy AR Metge BJ Alsheikh HA Shevde LA Samant RS. Ribosome Biogenesis: A Central Player in Cancer Metastasis and Therapeutic Resistance. Cancer Res. (2022) 82:2344–53. doi: 10.1158/0008-5472.CAN-21-4087 PMC9256764 35303060

[62] Cubillos-Ruiz JR Bettigole SE Glimcher LH. Tumorigenic and Immunosuppressive Effects of Endoplasmic Reticulum Stress in Cancer. Cell. (2017) 168:692–706. doi: 10.1016/j.cell.2016.12.004 28187289 PMC5333759

[63] Mills EW Green R. Ribosomopathies: There's strength in numbers. Science. (2017) 358 608,1-8. doi: 10.1126/science.aan2755 29097519

[64] Walter P Ron D. The unfolded protein response: from stress pathway to homeostatic regulation. Science. (2011) 334:1081–86. doi: 10.1126/science.1209038 22116877

[65] Hartl FU Hayer-Hartl M. Converging concepts of protein folding *in vitro* and *in vivo* . Nat Struct Mol Biol. (2009) 16:574–81. doi: 10.1038/nsmb.1591 19491934

[66] Truitt ML Ruggero D. New frontiers in translational control of the cancer genome. Nat Rev Cancer. (2016) 16:288–304. doi: 10.1038/nrc.2016.27 27112207 PMC5491099

[67] Liang J Lei K Liang R Huang J Tan B Lin H. Single-cell RNA sequencing reveals the MIF-ACKR3 receptor-ligand interaction between iCAFs and tumor cells in esophageal squamous cell carcinoma. Cell Signal. (2024) 117:111093. doi: 10.1016/j.cellsig.2024.111093 38336189

[68] Rani A Greenlaw R Smith RA Galustian C. HES1 in immunity and cancer. Cytokine Growth Factor Rev. (2016) 30:113–17. doi: 10.1016/j.cytogfr.2016.03.010 27066918

[69] Liu ZH Dai XM Du B. Hes1: a key role in stemness, metastasis and multidrug resistance. Cancer Biol Ther. (2015) 16:353–59. doi: 10.1080/15384047.2015.1016662 PMC4622741 25781910

[70] Murray IA Patterson AD Perdew GH. Aryl hydrocarbon receptor ligands in cancer: friend and foe. Nat Rev Cancer. (2014) 14:801–14. doi: 10.1038/nrc3846 PMC4401080 25568920

[71] Ma S Sa L Zhang J Jiang K Mi B Shan L. KDELR2 as a diagnostic and prognostic biomarker of bladder urothelial carcinoma and its correlation with immune infiltration. Genet Mol Biol. (2023) 46:e20230002. doi: 10.1590/1678-4685-GMB-2023-0002 37791813 PMC10548500

[72] Mao H Nian J Wang Z Li X Huang C. KDELR2 is an unfavorable prognostic biomarker and regulates CCND1 to promote tumor progression in glioma. Pathol Res Pract. (2020) 216:152996. doi: 10.1016/j.prp.2020.152996 32534703

[73] Oh MH Sun IH Zhao L Leone RD Sun IM Xu W. Targeting glutamine metabolism enhances tumor-specific immunity by modulating suppressive myeloid cells. J Clin Invest. (2020) 130:3865–84. doi: 10.1172/JCI131859 PMC7324212 32324593

[74] Yoshihara K Shahmoradgoli M Martinez E Vegesna R Kim H Torres-Garcia W. Inferring tumour purity and stromal and immune cell admixture from expression data. Nat Commun. (2013) 4:2612. doi: 10.1038/ncomms3612 24113773 PMC3826632

[75] Mantovani A Biswas SK Galdiero MR Sica A Locati M. Macrophage plasticity and polarization in tissue repair and remodelling. J Pathol. (2013) 229:176–85. doi: 10.1002/path.4133 23096265

[76] Qian BZ Pollard JW. Macrophage diversity enhances tumor progression and metastasis. Cell. (2010) 141:39–51. doi: 10.1016/j.cell.2010.03.014 20371344 PMC4994190

[77] Mata-Haro V Cekic C Martin M Chilton PM Casella CR Mitchell TC. The vaccine adjuvant monophosphoryl lipid A as a TRIF-biased agonist of TLR4. Science. (2007) 316:1628–32. doi: 10.1126/science.1138963 17569868

[78] Ries CH Cannarile MA Hoves S Benz J Wartha K Runza V. Targeting tumor-associated macrophages with anti-CSF-1R antibody reveals a strategy for cancer therapy. Cancer Cell. (2014) 25:846–59. doi: 10.1016/j.ccr.2014.05.016 24898549

[79] Pyonteck SM Akkari L Schuhmacher AJ Bowman RL Sevenich L Quail DF. CSF-1R inhibition alters macrophage polarization and blocks glioma progression. Nat Med. (2013) 19:1264–72. doi: 10.1038/nm.3337 PMC3840724 24056773

[80] Kortylewski M Kujawski M Wang T Wei S Zhang S Pilon-Thomas S. Inhibiting Stat3 signaling in the hematopoietic system elicits multicomponent antitumor immunity. Nat Med. (2005) 11:1314–21. doi: 10.1038/nm1325 16288283

[81] Kaneda MM Messer KS Ralainirina N Li H Leem CJ Gorjestani S. PI3Kgamma is a molecular switch that controls immune suppression. Nature. (2016) 539:437–42. doi: 10.1038/nature19834 PMC5479689 27642729

[82] Trinchieri G. Interleukin-12 and the regulation of innate resistance and adaptive immunity. Nat Rev Immunol. (2003) 3:133–46. doi: 10.1038/nri1001 12563297

