# Supplementary figures and images for: A new perspective on macrophage-targeted drug research: the potential of KDELR2 in bladder cancer immunotherapy
Source: Front Immunol. 2024 Dec 3;15:1485109. doi: 10.3389/fimmu.2024.1485109 (PMC11649672; doi:10.3389/fimmu.2024.1485109)

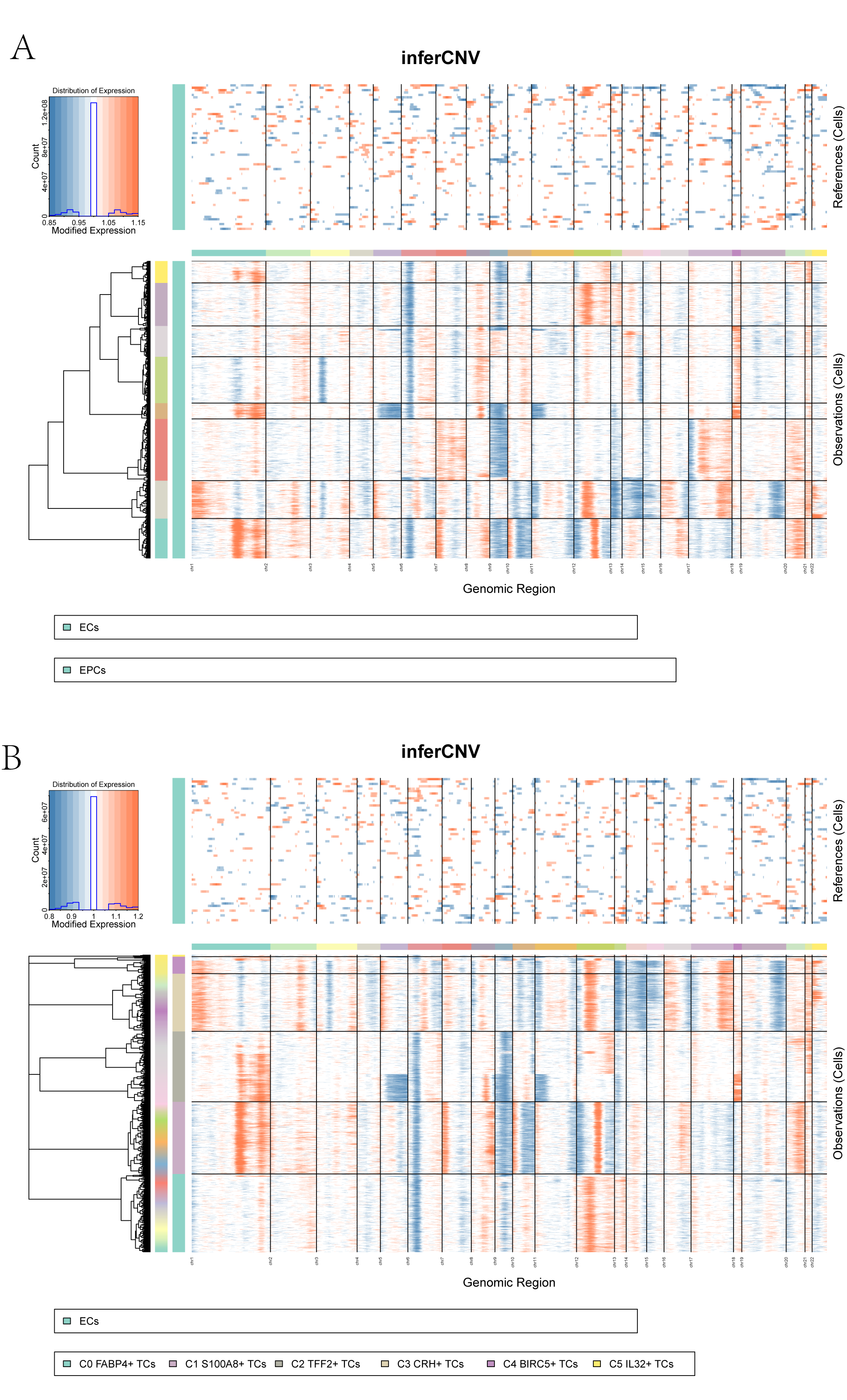

Supplement: Supplementary Figure 1 — The heatmaps showed the scores of CNV for ECs, EPCs, and six tumor cell clusters. (A) The heatmaps showed the scores of CNV for ECs and EPCs. (B) The heatmaps showed the scores of CNV for ECs and six tumor cell clusters. [file Image1.tif]
